# Interphase cell morphology defines the mode, symmetry, and outcome of mitosis

**DOI:** 10.1126/science.adu9628

**Published:** 2025-05-01

**Authors:** Holly E. Lovegrove, Georgia E. Hulmes, Sabrina Ghadaouia, Christopher Revell, Marta Giralt-Pujol, Zain Alhashem, Andreia Pena, Damian D. Nogare, Ellen Appleton, Guilherme Costa, Richard L. Mort, Christoph Ballestrem, Gareth W. Jones, Cerys S. Manning, Ajay B. Chitnis, Claudio A. Franco, Claudia Linker, Katie Bentley, Shane P. Herbert

**Affiliations:** 1 Faculty of Biology, Medicine and Health, University of Manchester; Oxford Road, Manchester, UK; 2 Department of Molecular, Cellular and Developmental Biology; Yale University; New Haven, USA; 3 Cellular Adaptive Behaviour Lab, Francis Crick Institute; London, UK; 4 Department of Mathematics, University of Manchester; Oxford Road, Manchester, UK; 5 Department of Biomedical Engineering and the Biological Design Center, Boston University; Boston, Massachusetts, USA; 6 Astbury Centre for Structural Molecular Biology, University of Leeds; Leeds, UK; 7 Randall Centre for Cell and Molecular Biophysics, Guy’s Campus, King’s College London; London, UK; 8 Instituto de Medicina Molecular João Lobo Antunes, Faculdade de Medicina, Universidade de Lisboa; Lisboa, Portugal; 9 Division of Developmental Biology, Eunice Kennedy Shriver National Institute of Child Health and Human Development; Bethesda, MD, USA; 10 Bioimage Analysis Infrastructure Unit, National Facility for Data Handling and Analysis, Human Technopole; Milan, Italy; 11 Wellcome-Wolfson Institute for Experimental Medicine, Queen’s University; Belfast, UK; 12 Division of Biomedical and Life Sciences, Faculty of Health and Medicine, Lancaster University; Lancaster, UK; 13 Universidade Católica Portuguesa, Católica Medical School, Católica Biomedical Research Centre; Lisbon, Portugal; 14 Department of Informatics, King’s College London; London, UK

## Abstract

During tissue formation, dynamic cell shape changes drive morphogenesis, whilst asymmetric divisions create cellular diversity. Here we found that the shifts in cell morphology that shape tissues could concomitantly act as conserved instructive cues that trigger asymmetric division and direct core identity decisions underpinning tissue-building. We performed single-cell morphometric analyses of endothelial and other mesenchymal-like cells. Distinct morphological changes switched cells to an “isomorphic” mode of division, which preserved pre-mitotic morphology throughout mitosis. In isomorphic divisions, interphase morphology appeared to provide a geometric code defining mitotic symmetry, fate determinant partitioning, and daughter state. Rab4-positive endosomes recognized this code allowing them to respond to pre-mitotic morphology and segregate determinants accordingly. Thus, morphogenetic shape change sculpts tissue form whilst also generating cellular heterogeneity, thereby driving tissue assembly.

During embryonic morphogenesis, tissues are sculpted by the dynamic reorganisation of cells. Core to this process are the coordinated changes in individual cell shape that drive key morphogenetic events, including tissue movement, extension, folding and branching (1, 2). Tight control of cell shape remodelling is thus critical to defining final tissue architecture. Coincident with these morphogenetic changes, cells must also establish the cellular diversity that underpins tissue-building. As such, shifts in cell shape dynamics during morphogenesis often occur in parallel with a switch from symmetric divisions that promote growth to asymmetric cell divisions that generate tissue heterogeneity (3). For example, when endothelial or trunk neural crest cells initiate migration, like many other mesenchymal-like cell types in vivo, they stereotypically elongate (4, 5). Moreover, this morphological shift occurs concurrent with a switch to fate-determining asymmetric divisions (4–10). In the case of migrating endothelial cells, these divisions create daughters with differential leader-follower identity that coordinate blood vessel morphogenesis (4, 7). In contrast, asymmetric trunk neural crest cell division creates daughters of distinct sympathetic ganglia or Schwann progenitor cell fate (9). How cells achieve spatiotemporally coupling of key morphogenetic events with a switch to asymmetric division is unclear, and if shifts in cell morphology have any influence on these decisions is unknown.

Cell division itself also triggers dramatic changes in cell morphology (11, 12). Upon entering mitosis, animal cells typically round up following global reorganisation of the interphase cytoskeleton, leading to a state of high cortical tension and low substrate adhesion. Mitotic rounding is highly conserved in metazoan cells and ensures high-fidelity symmetric segregation of genetic material, by facilitating chromosome capture and generating sufficient space for spindle formation (13–16). Consequently, failure to round often promotes spindle pole splitting, chromosome mis-segregation and mitotic cell death. In addition, mitotic rounding creates a uniform sphere that facilitates robust central positioning of the mitotic spindle that aids generation of equal sized daughter cells. As such, rounding also promotes equal partitioning of non-genetic cellular components, including organelles, that are often fragmented and/or homogenously distributed in mitotic cells and symmetrically inherited by equally sized daughters (17–19). Manipulation of mitotic rounding would thus appear an elegant means to switch cells from a symmetric to asymmetric mode of division. Whether it is possible for cells to tune the extent of their mitotic rounding, and how this would impact mitotic symmetry and/or daughter cell fate remain unexplored.

## A switch to isomorphic division breaks the symmetry of daughter cell size

**Fig 1:**
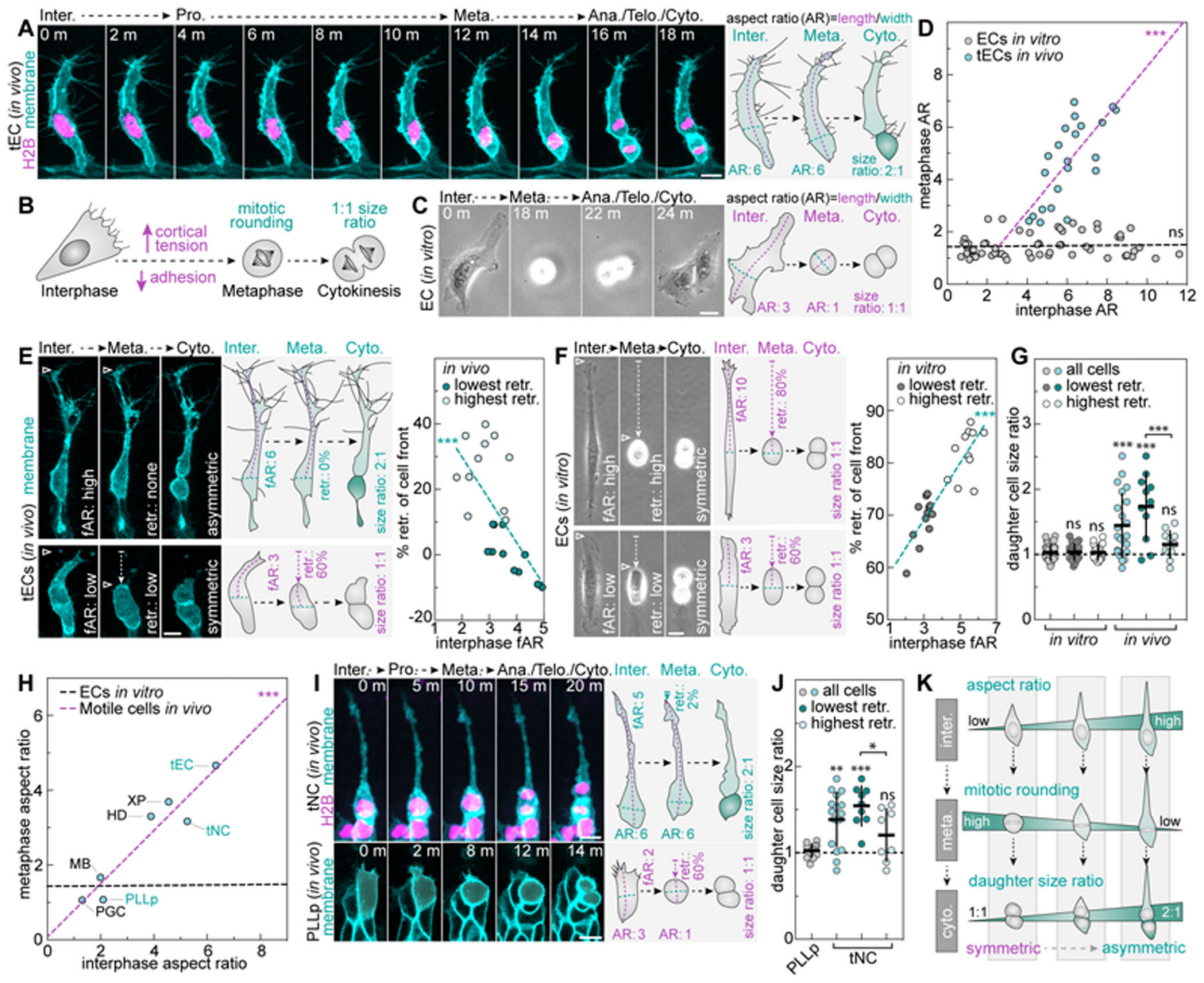
A switch to isomorphic division breaks the symmetry of daughter cell size. (**A**) Images of a mosaic Cerulean-H2B-/lyn-mCherry-expressing zebrafish tEC undergoing mitosis. Schematic shows aspect ratio (AR) and daughter size ratios. (**B**) Classical cell division. (**C**) Images of a mitotic human EC in 2D culture. (**D**) AR in interphase versus metaphase for tECs in vivo (magenta line; *n*=22) and ECs in vitro (black line; *n*=60). (**E,F**) Images of mosaic lyn-mCherry-expressing zebrafish tECs (E) or human ECs on micropatterned fibronectin lines of varying width (F) undergoing mitosis (arrowheads indicate cell front; arrows indicate retraction of cell front). Schematics show front AR (fAR), percentage retraction of the cell front and daughter size ratios. Plots compare interphase fAR versus mitotic retraction (retr.) of the cell front (light or dark data points indicate the 50% highest or 50% lowest retracting cells, respectively; *n*=22 (E), *n*=23 (F)). (**G**) Daughter cell size ratio of all, the 50% highest retracting or 50% lowest retracting ECs in vitro or tECs in vivo (*n*=41 in vitro, *n*=22 in vivo). (**H**) Interphase versus metaphase AR for the indicated mesenchymal-like cell types in vivo (magenta line; black line indicates ECs in vitro from (D); *n*=7 cell types (as listed in fig. S2F)). (**I**) Images of a H2B-mCherry/GLYPI-EGFP-labelled trunk neural crest (tNC) cell and a lyn-EGFP labelled posterior lateral line primodium (PLLp) cell undergoing mitosis. Schematics show AR change, fAR, percentage retraction of the cell front and daughter size ratios. (**J**) Daughter cell size ratio of all PLLp cells, the 50% highest- or 50% lowest-retracting tNCs. (*n*=14 PLLp, *n*=17 tNC). (**K**) Relationship between isomorphic division and asymmetry in daughter size. Data are mean ± s.d. (G,J). ***P=<0.0008, **P=<0.0014, *P=<0.00366, ns *P*=>0. 3462, versus either all cells in vitro (G) or all PLLp cells (J), unless indicated by bracket. Two-tailed Pearson’s correlation coefficient for D (*r*=0.7334), E (*r*=-0.6729), F (*r*=0.8884) and H (*r*=0.9544). Unpaired ANOVA and Tukey’s multiple comparison test for G and J. For A,C,E,F and I, scale bars, 10µm.

New blood vessel morphogenesis by the process of angiogenesis involves dramatic cell shape remodelling. Initially, quiescent endothelial cells (ECs) in a parental vessel are specified to acquire motile ”tip” EC (tEC) identity, which triggers a substantial shift in morphology from a stable cobblestone-like form to an invasive elongated mesenchymal-like state that directs neovessel branching (10, 20, 21). Concomitant with this morphological switch, tECs in zebrafish embryos adopt asymmetric cell divisions that create daughters of distinct leading “tip” or trailing “stalk” cell state (4, 6, 10). As such, this transition to asymmetric division coordinates collective cell migration. To determine if initial stereotyped shifts in tEC morphology could impact this switch from mitotic symmetry to an asymmetric division outcome, we performed a detailed morphometric analysis of mosaic tEC shape dynamics during zebrafish intersegmental vessel formation (fig. S1A). This single-cell analysis revealed that mitotic tECs adopt a mode of cell division that uncharacteristically preserved interphase morphology throughout mitosis (Fig. 1A; movie S1). Upon mitotic entry, metazoan cells typically adopt a spherical geometry, following global reorganization of the interphase cytoskeleton (11, 12) (Fig. 1B,C; movie S2). Mitotic rounding is considered critical for high-fidelity division (13, 14, 16). However, extraction of key morphodynamic parameters (such as aspect ratio (AR); fig. S1A) confirmed that dividing tECs frequently evaded dramatic mitotic shape change and retained interphase-like morphological metrics (Fig. 1D; fig. S1B). We termed this mode of division “isomorphic” because cells retain interphase morphology throughout mitosis. Human ECs in 2D culture, in contrast, exhibited classic mitotic rounding and robustly adopted a spherical geometry in metaphase (AR ~1) regardless of their interphase geometry (Fig. 1C-D; fig. S1B; movie S2). Mitotic tECs in the mouse retina also adopted isomorphic division and retained an elongated geometry (fig. S2A). Thus, ECs in vivo and in 2D culture in vitro exhibit stark differences in mode of division.

Comparative analyses of interphase versus mitotic morphodynamics revealed that a shift to isomorphic division was linked to stereotyped morphogenetic shape change in interphase. When newly specified tECs exit a parental vessel, they elongate their leading-edge as they initiate migration, similar to most motile mesenchymal-like cells in vivo (4, 22, 23). Initiation of tEC motility thus corresponds with pronounced cell protrusion (as defined by the morphometric readout ‘front’ AR (fAR; length of the cell front divided by the width of the widest point); fig. S1A and fig. S2B-D).This stereotyped tEC morphological change was also tightly associated with a shift to isomorphic division (Fig. 1E; fig. S1C,D; movie S3 and movie S4). Unlike other interphase morphometric parameters, which were poorly linked to evasion of mitotic rounding, tEC elongation and high interphase AR/fAR values were uniquely predictive of isomorphic divisions (Fig. 1E; fig. S1C,D). Indeed, the most protrusive tECs exhibited no cell retraction and rounding during division, and even continued to elongate in mitosis (Fig. 1E; movie S3). In contrast, the relationship between interphase protrusion and the mitotic shape change was inverted in in vitro cells that exhibit mitotic rounding because highly elongated cells underwent greater retraction in mitosis to assume a spherical geometry (Fig. 1F; movie S5 and movie S6).

Thus, stereotyped morphogenetic shape change in vivo is linked to a switch in the mode of cell division.

As tECs initiate migration, transition to asymmetric division generates daughters of unequal size that acquire distinct leader-follower behaviours and coordinate angiogenesis (4, 7, 10) (Fig. 1A). To determine if tEC migration, elongation and a shift to isomorphic division were coupled to a switch to asymmetric division outcome, we quantified post-mitotic size. Indeed, only highly protrusive tECs (that exhibited little to no mitotic rounding and adopted isomorphic mitosis) triggered asymmetric divisions that generated daughters of distinct size (Fig. 1G; Fig. S2E). In contrast, less-motile tECs that experienced greater mitotic rounding generated near-symmetric-sized daughters (Fig. 1G). Distinct morphogenetic changes thus appear to shift cells to isomorphic divisions that break the symmetry of daughter cell size.

Almost all mesenchymal-like cells protrude their leading-edge as they migrate (23). Indeed, expansion of morphometric analyses to other motile cell types in vivo confirmed conservation of this interrelationship between cell elongation, isomorphic division, and symmetry of mitosis (Fig. 1H-J; fig. S2F,G). Like tECs, other highly elongated motile cell types were consistently isomorphic in division and exhibited a tight correlation between interphase and metaphase morphological metrics (Fig. 1H). The observation that protrusive trunk neural crest (tNC) leader cells adopted isomorphic divisions (Fig. 1H,I fig. S2G; movie S7) was particularly notable because morphogenetic tNCs also transition to asymmetric cell divisions as they migrate (5, 9). Analogous to tEC mitosis, asymmetric tNC divisions also create daughters of distinct size with differential sympathetic ganglia or Schwann cell fate (9). Moreover, only isomorphic tNC divisions that evaded mitotic rounding generated these asymmetric-sized daughters (Fig. 1J), indicating a conserved role for a shift to isomorphic division in triggering asymmetric division.

In contrast, less-protrusive motile cell types, such as posterior lateral line primordium (PLLp) leader cells, robustly underwent mitotic rounding and consistently generated near-symmetric sized daughters (Fig. 1H-J; movie S8). Thus interphase cell morphology may play a conserved role in precise tuning of the mode and symmetry of mitosis (Fig. 1K).

## Interphase morphometric cues tune the mode and symmetry of mitosis

**Fig 2:**
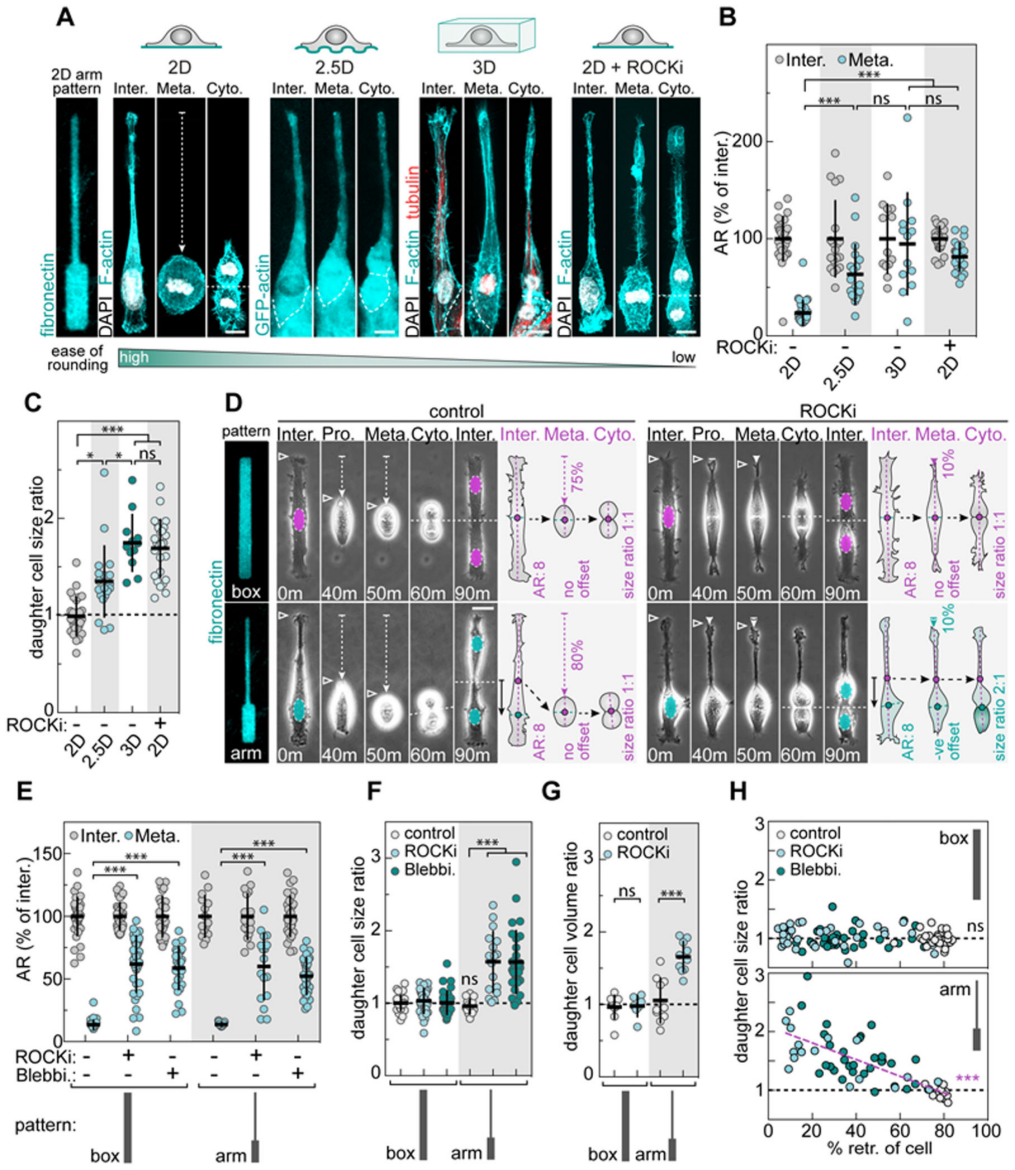
Interphase morphometric cues tune the switch to isomorphic division. (**A**) Human ECs seeded on 2D micropatterned fibronectin shapes (in the presence or absence of ROCKi), on a feeder layer of human dermal fibroblasts (2.5D), or in 3D fibrin gels. ECs were labelled with DAPI and rhodamine-phalloidin (2D and 3D), immunostained for tubulin (3D) or expressed GFP-actin (2.5D; white line indicates cell-cell boundaries; arrow indicates retraction of cell front). (**B,C**) ARs in interphase and metaphase (B) or daughter cell size ratios (C) of ECs cultured as in panel A (*n*=30 2D, *n*=19 2.5D, *n*=13 3D, *n*=28 2D + ROCKi). (**D**) Human ECs seeded on fibronectin shapes undergoing mitosis in the presence or absence of ROCKi (arrowheads, dashed arrows and dashed lines indicate cell front, retraction of cell front and cell-cell boundary, respectively; nuclei are pseudocolored). Schematics show constant AR, offset of the nuclei and metaphase plate on arm patterns (arrow), percentage retraction of the cell front and daughter size ratios. (**E,F**) Interphase and metaphase ARs (E) or daughter size ratios (F) for ECs seeded on box or arm-shapes in the presence or absence of ROCKi or blebbistatin (Blebbi.; *n*=36 control box, *n*=36 ROCKi box, *n*=27 Blebbi. Box, *n*=15 control arm, *n*=17 ROCKi arm, *n*=30 Blebbi. Arm). (**G**) Post-mitotic daughter volume ratio for ECs seeded on box or arm-shapes in the presence or absence of ROCKi (*n*=10 control box, *n*=7 ROCKi box, *n*=10 control arm, *n*=10 ROCKi arm). (**H**) Percentage retraction of the cell versus daughter cell size ratio for control, ROCKi-treated and Blebbi.-treated ECs seeded on box or arm-shapes (black lines indicate symmetric daughter size; magenta line indicates ECs on arm patterns; sample sizes as in F. Data are mean ± s.d. (B,C,E,F and G). ***P=<0.0003, *P=<0.0262, ns *P*=>0.5577, versus control box in F, unless indicated by bracket. Unpaired Kruskal–Wallis test and Dunn multiple comparison test for B, C, E and F. Two-tailed unpaired t-test for G. Two-tailed Pearson’s correlation coefficient for H (*r*=-0.7504). For A and D, scale bars, 10µm.

It was unclear why ECs in 2D culture consistently underwent mitotic rounding but switched to isomorphic division in vivo. Dividing cells in vitro find it harder to round in 3D matrix versus on 2D substrates, likely owing to increased cell adhesion and/or confinement (24, 25). Human ECs that normally round in 2D consistently shifted to fully isomorphic and asymmetric divisions upon exposure to increasingly complex 2.5D or 3D microenvironments (Fig. 2A-C). Hence, a switch to isomorphic division appears an inherent feature of ECs in environments that pose sufficient challenge to mitotic rounding. To confirm this concept, human ECs were seeded on 2D micropatterned fibronectin shapes (26) that predictably modulate cell morphology to accurately mimic 2.5D and 3D cells (Fig. 2A; fig. S3A). These micropatterned cells stereotypically rounded in division, aligned their mitotic spindle perpendicular to the adhesive micropattern (27, 28) and created symmetric sized daughter cells (Fig. 2A-C; fig. S3B). However, these cells could be shifted to robust asymmetric isomorphic divisions upon disruption of mitotic rounding using Y-27632 (ROCKi), which inhibits ROCK and perturbs mitotic cortical contractility (29) (Fig. 2A-C; fig. S3B). Thus, tuning the ease of mitotic rounding can determine the mode and symmetry of EC division.

A core feature of isomorphic division is that cells uncharacteristically retain their interphase morphology throughout mitosis, suggesting that pre-mitotic cell morphology can then instruct mitotic events. Indeed, ECs and tNCs that divide asymmetrically, both in vivo and in vitro, consistently exhibit a similar polarized interphase morphology characteristic of highly motile cells, with clear front-rear shape asymmetry and a rearward nucleus positioning (30) (Fig. 1A,E,I,K and Fig. 2A). To test if this pre-mitotic morphology indeed defines mitotic symmetry, we exploited our micropatterning-mediated model of isomorphic division (Fig. 2A). Human ECs were seeded on 2D micropatterned fibronectin shapes that either recapitulated the polarised morphology of ECs in vivo and in vitro (Fig. 2A; arm pattern) or induced a symmetric shape (box pattern), creating either an offset or equatorial nucleus positioning, respectively (Fig. 2D). In control ECs that fully rounded in mitosis, interphase shape and nucleus placement had no impact on division outcome because rounding efficiently centralized both the metaphase plate and cleavage furrow to generate symmetric-sized daughters (Fig. 2D-G; movie S9). In contrast, if mitotic rounding was perturbed upon inhibition of ROCK (ROCKi) or myosin II (blebbistatin) activity (29) (Fig. 2D,E; fig. S4A,B), interphase geometry defined the symmetry of division (Fig. 2D-G; movie S10). In particular, on arm patterns, an asymmetric interphase morphology generated significant asymmetries in daughter size (arm pattern; Fig. 2D,F,G; fig. S4A,C,D; movie S10). Moreover, the magnitude of this mitotic asymmetry was tightly dependent on the level of cell retraction and rounding experienced (Fig. 2H), confirming that a shift to isomorphic division triggered this phenomenon. Conversely, on box patterns, near-symmetric interphase geometry robustly generated symmetric-sized daughters, even if mitotic rounding was impaired and ECs experienced little rounding (Fig. 2D-H; fig. S4A,C,D; movie S10). Thus, interphase cell morphology precisely tunes the symmetry of mitosis in isomorphic divisions.

**Fig 3:**
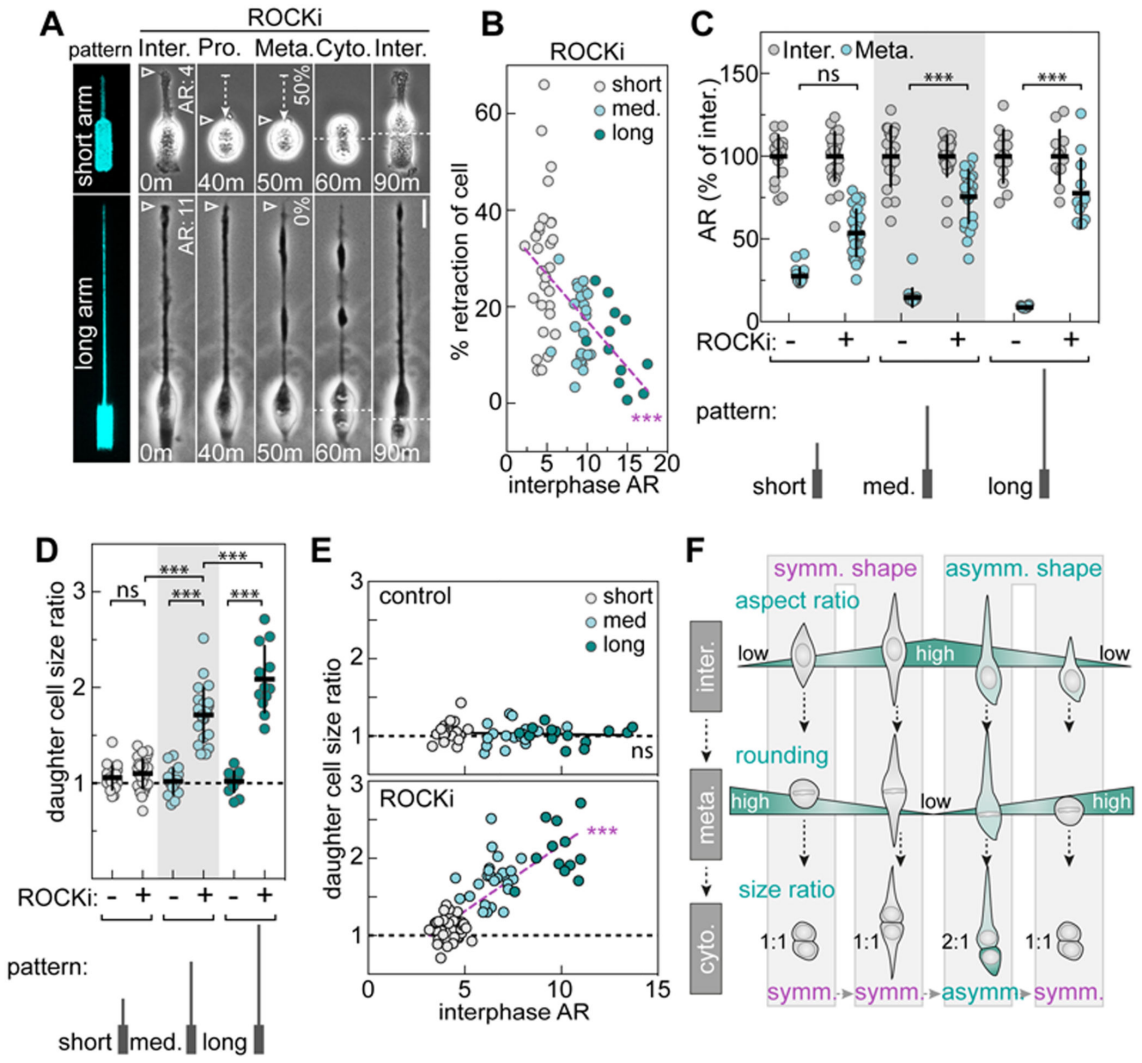
Interphase cell length tunes the switch to isomorphic division. (**A**) ROCKi-treated human ECs seeded on the indicated micropatterned fibronectin shapes undergoing mitosis (arrowheads, dashed arrows and dashed lines indicate cell front, retraction of cell front and cell-cell boundary, respectively). (**B**) Interphase AR versus percentage retraction of the cell for ROCKi-treated ECs seeded on short, medium (med.) or long arm-shapes (*n*=29 short, *n*=24 med., *n*=12 long). (**C**) Interphase and metaphase ARs for ECs seeded on short, med. or long arm-shapes in the presence or absence of ROCKi (*n*=20 short, *n*=29 ROCKi short, *n*=20 med., *n*=24 ROCKi med., *n*=14 long, *n*=12 ROCKi long). (**D**) Post-mitotic daughter size ratio for ECs seeded on short, med. or long arm-shapes in the presence or absence of ROCKi (*n*=21 short, *n*=34 ROCKi short, *n*=18 med., *n*=25 ROCKi med., *n*=14 long, *n*=12 ROCKi long). (**E**) Interphase AR versus daughter cell size ratio for ECs seeded on short, med. or long arm-shapes in the presence or absence of ROCKi (black lines indicate symmetric daughter size; magenta line indicates ECs treated with ROCKi; sample sizes as in D; two-tailed Pearson's correlation coefficient). (**F**) Relationship between shifts in interphase morphology, isomorphic division and daughter size asymmetry. Data are mean ± s.d. (C and D). ***P=<0.0001, ns *P*=>0.0522. Two-tailed Pearson’s correlation coefficient for B (*r*=-0.5314) and E (*r*=0.8050). Unpaired Kruskal–Wallis test and Dunn multiple comparison test for C. Unpaired ANOVA and Tukey’s multiple comparison test for D. For A, scale bar, 10µm.

Morphometric analysis in vivo additionally suggested that cell elongation could induce isomorphic division (Fig. 1E). Exploiting our micropatterns, we confirmed the impact of cell length on this switch in division mode. Upon seeding ECs on 2D micropatterned arm shapes of differing lengths we noted that cells with shorter protrusions consistently underwent mitotic rounding and generated daughters of symmetric size, even in the presence of ROCKi (Fig. 3A-E). In contrast, as cells increase in length, they exhibited both significantly reduced mitotic rounding and significantly increased daughter size asymmetry (Fig. 3A-D). Consequently, daughter cell size ratio was tightly dependent on interphase AR in isomorphic divisions (Fig. 3E), which suggests that cell elongation is a key trigger for the switch to isomorphic division. Moreover, exploration of mitosis on other micropatterned shapes verified that increased length was insufficient to trigger symmetry-breaking of isomorphic division alone because this only occurred with an additional asymmetry in morphology (Fig. 3A,D,E; fig. S5A-C). Thus, an elongated and asymmetric interphase cell morphology acts as a geometric code that defines the mode and symmetry of mitosis (Fig. 3F).

**Fig 4:**
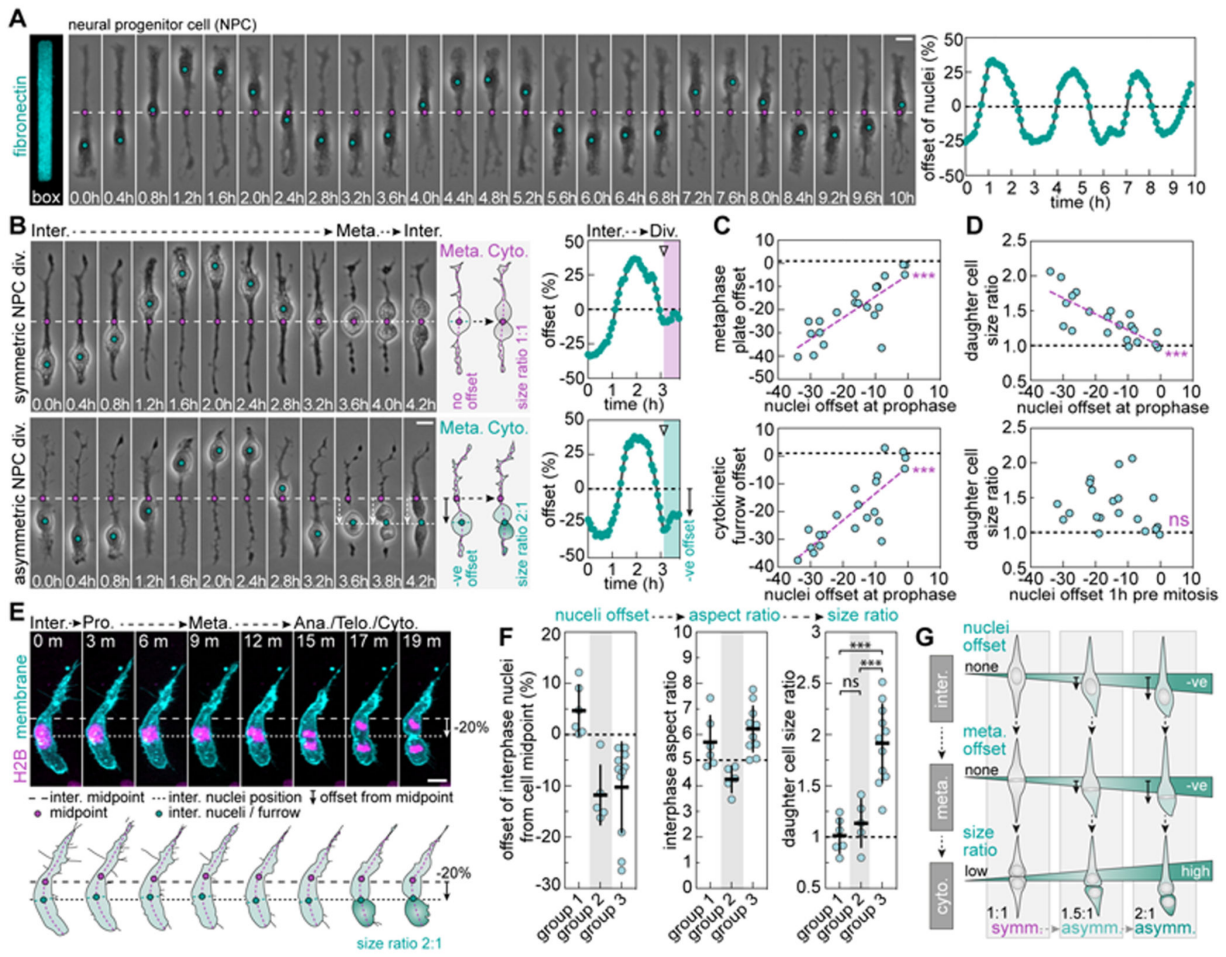
Interphase nuclei positioning defines the symmetry of isomorphic division. (**A**) Mouse neural progenitor cell (NPC) seeded on a box shape (magenta dots/white line indicate NPC midpoint, cyan dots indicate nuclei position). Plot shows nuclei offset from the NPC midpoint. (**B**) ROCKi-treated NPCs seeded on box shapes undergoing mitosis with either an equatorial (symmetric NPC div.) or polar (asymmetric NPC div.) prophase nucleus (magenta dots/course dashed white lines indicate NPC midpoint, cyan dots indicate nuclei position, arrows indicate offset from the NPC midpoint). Schematics show daughter size ratios. Plots show offset from the midpoint of respective NPC nuclei over time, as well as offset of the metaphase plate and cytokinetic furrow in division (Div.; arrowhead and shaded area indicate mitosis, arrow indicates offset of the cleavage furrow). (**C**) Offset of the NPC nuclei at prophase versus either offset of the metaphase plate or offset of the cleavage furrow for NPCs in the presence of ROCKi (*n*=21). (**D**) Offset of the NPC nuclei either at prophase or 1h prior to prophase versus daughter cell size ratio for NPCs in the presence of ROCKi (*n*=21). (**E**) Mosaic Cerulean-H2B-/lyn-mCherry-expressing tEC undergoing mitosis (fine dashed line indicates interphase nuclei position; coarse dashed line indicates midpoint of cell length; arrow indicates nuclei offset from midpoint as a % of total cell length). Schematic shows these morphological landmarks throughout mitosis and daughter size ratio. (**F**) Either the offset of interphase nuclei from the cell midpoint, interphase ARs or daughter cell size ratios for tECs in vivo split into three groups. Group 1: tECs with nuclei offset ≥0; Group 2: tECs with nuclei offset ≤0 and AR ≤5; Group 3: tECs with nuclei offset ≤0 and AR ≥5 (*n*=6 Group 1, *n*=5 Group 2, *n*=11 Group 3). (**G**) Relationship between nuclei positioning and daughter size asymmetry. Data are mean ± s.d. (F). ***P=<0.0005, ns *P*=>0.3808. Two-tailed Pearson's correlation coefficient for C (*r*=0.7983 for metaphase plate, *r*=0.8181 for cleavage furrow) and D (*r*=-0.7772). For A,B and E, scale bars, 10µm.

Further quantification of micropatterned cells revealed that interphase nuclei positioning was highly predictive of subsequent metaphase plate and cleavage furrow positioning in isomorphic divisions (fig. S6A), both of which ultimately determined daughter size symmetry. On asymmetric arm micropatterns, initial biased proximal positioning of interphase cell nuclei was mirrored by biased mitotic positioning of both the metaphase plate and cleavage furrow in isomorphic divisions. (fig. S6A; arm pattern + ROCKi/Blebbi). In contrast, mitotic rounding abolished any biased positioning in control cells (fig. S6A-G). Indeed, when division was isomorphic, interphase nuclei positioning was tightly correlated with metaphase plate and cleavage furrow positioning, as well as with subsequent asymmetry in daughter cell size (fig. S6H). Thus, asymmetry in interphase nuclei positioning may fundamentally define mitotic symmetry. However, the static nature of EC nuclei in these assays made it difficult to unpick the specific impact of nuclei positioning versus other potential geometric/mechanical effects of micropatterning. In contrast to the static nuclei of ECs, we found that that mouse neural progenitor cells (NPCs) exhibited highly oscillatory nuclei dynamics on micropatterns (Fig. 4A). Moreover, when NPC mitotic rounding was perturbed using ROCKi (fig. S6I), it was the final oscillatory position of nuclei prior to prophase entry that defined the symmetry of division (Fig. 4B). Whereas an equatorial nucleus just before prophase created symmetric-sized daughters, an offset polarised nucleus created post-mitotic asymmetry (Fig. 4B). Indeed, because nuclei positioning was broadly stochastic, isomorphic divisions introduced wide variance in metaphase plate and cleavage furrow positioning, as well as broad heterogeneity in post-mitotic daughter size (fig. S6J,K). This variance thus enabled the interrelationship between pre-prophase nuclei localization and either metaphase plate positioning, cleavage furrow positioning, or daughter size asymmetry to be fully confirmed (Fig. 4C,D). This association between nuclei positioning and post-mitotic size asymmetry was lost if nuclei localisation was recorded 1h prior to prophase entry, when nuclei were at a different point in their oscillation (Fig. 4D). Thus, nuclei positioning at prophase entry defines the symmetry of isomorphic division.

Taken together, these observations suggested that shifts in morphogenetic cell behaviour that impact interphase cell length and nuclei position are key morphological determinants of mitotic symmetry in vivo. Consistent with this hypothesis, biased proximal positioning of tEC nuclei in vivo was highly similar to micropatterned ECs in vitro (fig. S7A). In addition, pre-mitotic tEC nuclei positioning in vivo was also highly predictive of subsequent localisation of the metaphase plate and cleavage furrow (Fig. 4E; fig. S7B; movie S11), as observed in vitro (Fig. S6H). Live imaging of centrosome dynamics in vivo revealed that this interrelationship was driven by the known role of the nucleus in directing the movement and placement of duplicated/separated centrosomes to position the site of mitotic spindle formation (31) (fig. S7C-E). Consequently, knowledge of just interphase nuclei position and AR alone was sufficient to define mitotic tEC symmetry. In particular, tECs in which the interphase nucleus was not proximally biased generate symmetric sized daughters (group 1; Fig. 4F). tECs with proximally localised nuclei but low ARs, (and thus exhibited mitotic rounding), likewise divided symmetrically (group 2; Fig. 4F). Notably, only elongated tECs with both a high AR and proximal localised nuclei exhibited asymmetric division (group 3; Fig. 4F). Thus, distinct interphase morphogenetic changes driven by increased cell motility (high AR, proximal nuclei) act as key instructive cues that tune the mode of division and mitotic symmetry (Fig. 4G).

## Isomorphic division drives asymmetry in fate determinant inheritance and signalling state

**Fig 5:**
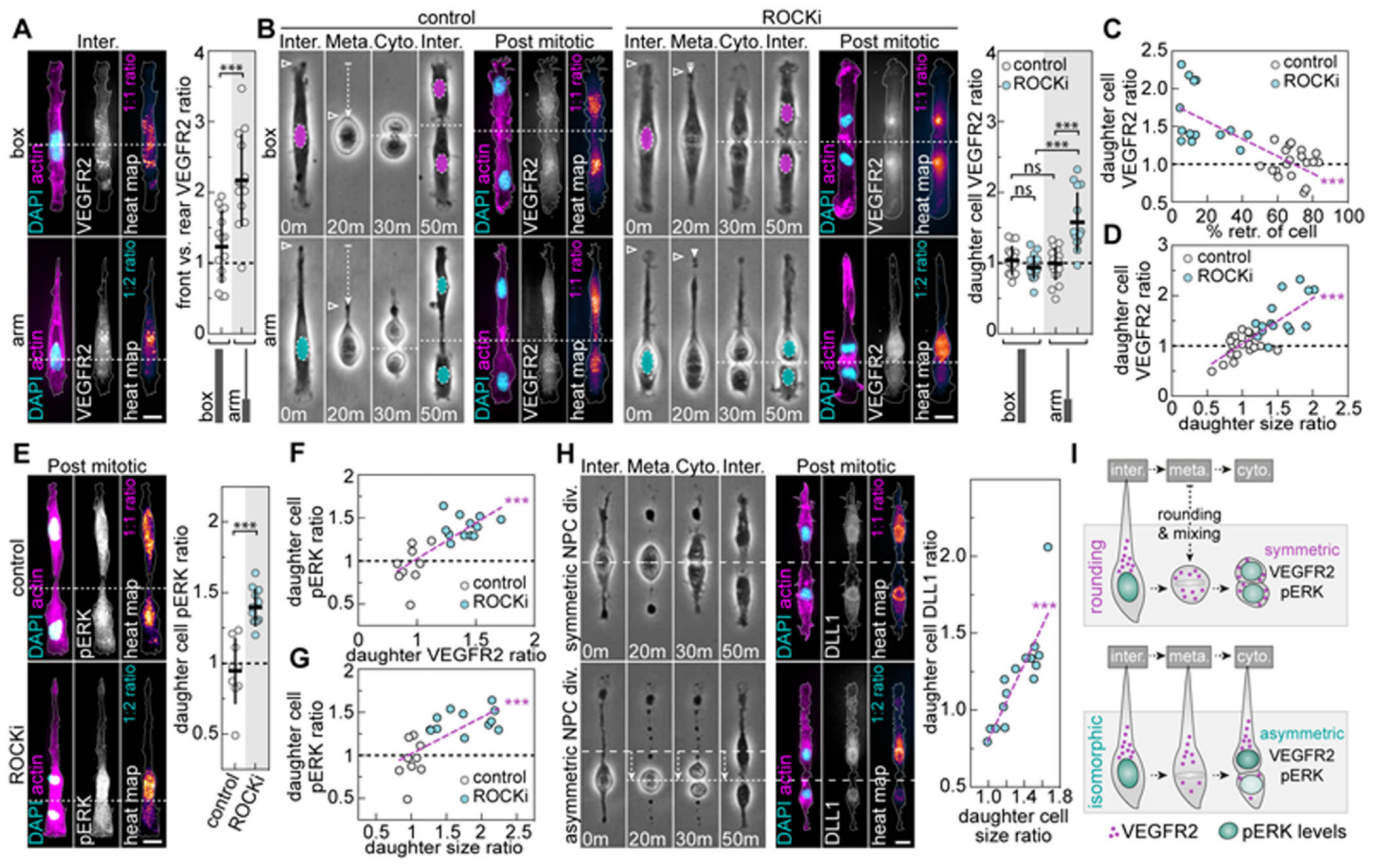
Isomorphic division promotes asymmetric partitioning of fate determinants. (**A**) ECs on arm/box shapes immunostained for VEGFR2 (line indicates nuclei position). Plot of the ratio of VEGFR2 fluorescence intensity forward versus rearward of nuclei (*n*=15 box, *n*=14 arm). (**B**) ECs on arm/box shapes undergoing mitosis then immunostained for VEGFR2 after division (arrowheads, arrows and lines indicate cell front, retraction and cell-cell boundary, respectively; nuclei are pseudocolored). Plot of the ratio of daughter VEGFR2 fluorescence intensity (*n*=18 control box, *n*=20 ROCKi box, *n*=20 control arm, *n*=15 ROCKi arm). (**C,D**) Percentage retraction of the cell front (C) or daughter size ratio (D) versus the ratio of daughter VEGFR2 fluorescence intensity for ECs seeded on arm-shapes (black lines indicate VEGFR2 symmetry; magenta lines indicate ECs on arm patterns; sample sizes as in B). (**E**) ECs on arm shapes immunostained for pERK after division (line indicates cell-cell boundary). Plot of the ratio of daughter pERK fluorescence intensity ECs (*n*=9 control, *n*=12 ROCKi). (**F,G**) Daughter VEGFR2 ratio (F) or daughter size ratio (G) versus the ratio of daughter pERK fluorescence intensity for ECs seeded on arm-shapes (black lines indicate pERK symmetry; magenta lines indicate ECs on arm patterns; sample sizes as in E). (**H**) NPCs on box shapes undergoing mitosis in the presence of ROCKi then immunostained for DLL1 after division (course dashed white lines either indicate pre-mitotic midpoint or post-mitotic cell-cell boundary, arrows indicate offset of cleavage furrow). Plot compares post-mitotic daughter size ratio versus the ratio of daughter DLL1 fluorescence intensity (*n*=15). (**I**) Relationship between isomorphic division and asymmetric partitioning of VEGFR2. Data are mean ± s.d. (A,B and E). ***P=<0.0003, ns *P*=>0.6437. Two-tailed unpaired t-test for A and E. Unpaired ANOVA and Tukey’s multiple comparison test for B. two-tailed Pearson's correlation coefficient for C (*r*=-0.7459),D (*r*=0.7761),F (*r*=0.7673), G (*r*=0.7124) and H (*r*=0.8589). For A,B,E and H, scale bars, 10µm.

Following asymmetric tEC division in vivo, differential inheritance of vascular endothelial growth factor receptor (VEGFR) signalling generates daughters of distinct leader-follower identity (4, 6). To identify if preceding shifts in interphase morphology underpin this asymmetry, we assessed whether manipulation of cellular morphologies that define isomorphic division and mitotic asymmetry could trigger predictable differential VEGFR partitioning. Indeed, simply shifting cells from symmetric to asymmetric micropatterns (box- versus arm-shaped patterns, respectively) induced front-rear polarity in the interphase vesicular localization of VEGFR-2 relative to the nucleus (Fig. 5A; fig. S8A,B). Despite this pre-polarization, if ECs underwent mitotic rounding, this vesicular VEGFR-2 compartment was efficiently mixed and symmetrically partitioned between daughters (Fig. 5B; fig. S8A,B), consistent with a role for rounding in promoting mixing and symmetric partitioning of cellular components (12, 13, 17–19, 32). In contrast, this interphase front-rear pre-polarization was maintained throughout division if rounding was perturbed, resulting in robust asymmetric partitioning of VEGFR-2 (Fig. 5B; fig. S8C,D). Differential VEGFR-2 inheritance was tightly associated with the magnitude of mitotic rounding (Fig. 5C) and was linked to final asymmetry in daughter cell size (Fig. 5D). Distinct post-mitotic VEGFR-2 levels ultimately drive differential leader-follower identity by creating differences in daughter ERK activity (4, 6, 7). Manipulation of interphase morphology indeed created comparable asymmetry in daughter pERK levels (Fig. 5E) that were tightly linked to post-mitotic asymmetry in VEGFR-2 inheritance and differential daughter size (Fig. 5F,G).

However, subtle differences in the interphase subcellular distribution of other identity determinants had a profound impact on their mitotic partitioning. Unlike VEGFR-2, the interphase localization of another key determinant of tip-stalk identity, the Notch ligand DLL4 (33, 34), was not influenced by cell geometry (fig. S8E-H). DLL4 was localized in a peri-nuclear membrane compartment that did not exhibit any front-rear polarity relative to the nucleus, even when the nucleus was offset (fig. S8E). Consequently, DLL4 was robustly symmetrically partitioned between daughters, independent of the level of mitotic rounding or daughter size asymmetry (fig. S8E-H). Thus, interphase front-rear polarization of identity determinants in specific vesicular compartments appears to be a key modulator of their asymmetric partitioning in isomorphic divisions. Moreover, these observations explain why differential tip-stalk behaviour of tEC daughters in vivo is initially driven by asymmetry in VEGFR, with DLL4 being redundant (4). Furthermore, these interphase geometric rules governing VEGFR-2 asymmetry were broadly applicable and could be exploited to force predictable asymmetric partitioning of fate determinants in other cell systems. For example, in NPCs the asymmetric partitioning of the post-mitotic neuronal fate determinant, Notch ligand DLL1 (35), was also triggered by disruption of mitotic rounding and asymmetry in daughter size (Fig. 5H). Thus, switches in cell morphology that tune isomorphic division promote asymmetric partitioning of key fate determinants (Fig. 5I).

**Fig 6:**
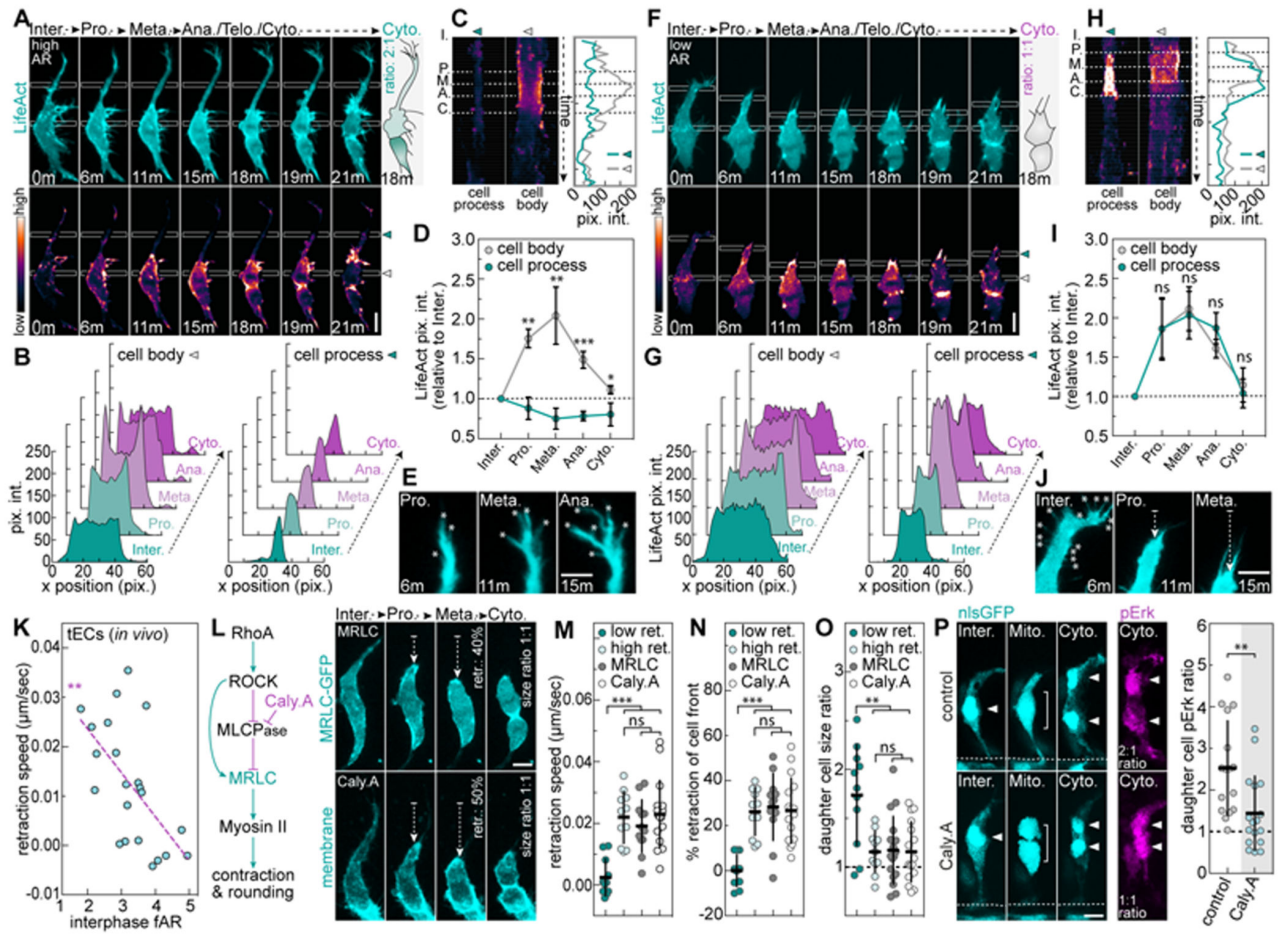
Modulation of isomorphic division dictates symmetry of mitosis in vivo. (**A,F**) Mosaic Lifeact-GFP-expressing zebrafish tECs of high AR (A) or low AR (F) undergoing mitosis. (**B,G**) Fluorescence intensity plots for the boxed areas in panel A (B) or F (G) at either the cell body or process during mitosis. (**C,H**) Kymographs generated using the boxed areas in panel A (C) or panel F (H) and intensity plots for cortical F-actin (grey arrowheads/line indicate cell body; cyan arrowheads/line indicate cell process). (**D,I**) Cortical intensity of F-actin at the cell body (grey line) or process (cyan line) of high AR tECs (D) or low AR tECs (I) during mitosis. (*n*=3). (**E,J**) Filopodia dynamics and cell retraction in mitosis taken from panel A (E) or panel F (J; asterisks indicate filopodia; arrows indicate retraction of cell front). (**K**) Interphase fAR versus speed of cell front retraction during tEC mitosis (*n*=22). (**L**) Schematic of RhoA-mediated actomyosin contractility during mitotic rounding. Mosaic MRLC-GFP-expressing tECs (MRLC) or mosaic lyn-mCherry-expressing tECs treated with calyculin A (Caly.A) undergoing mitosis (arrows indicate cell retraction). (**M,N,O**) Speed of cell retraction in mitosis (M), percentage retraction of the cell front (N) and daughter size ratio (O) in either Wt tECs (split into the 50% highest retracting or 50% lowest retracting cells), MRLC-GFP-expressing tECs or Caly.A-treated tECs (*n*=22 Wt, *n*=12 MRLC-GFP, *n*=16 Caly.A). (**P**) nlsGFP-expressing tECs undergoing mitosis in the absence or presence of Caly.A then immunostained for pErk (arrowheads and bracket indicate tEC nuclei and mitotic cells, respectively; line indicates the dorsal aorta). Plot of the ratio of daughter pErk fluorescence intensity in control and Caly.A-treated tECs (*n*=16 control, *n*=14 Caly.A). Data are mean ± s.d. ***P=<0.0005, **P=<0.0042, *P=0.0250, ns *P*=>0.6714. Two-tailed unpaired t-test for D and I. Two-tailed Pearson's correlation coefficient for K (*r*=-0.5971). Unpaired ANOVA and Tukey’s multiple comparison test for M,N and O. Two-tailed Mann-Whitney test for P. (D,L,M,N,O and P). For A,E,F,I,L, and P, scale bars, 10µm.

To determine if a shift to isomorphic division indeed underpins post-mitotic asymmetry in Vegfr activity in vivo, we aimed to define how mitotic shape could be manipulated during angiogenesis. First, we assessed how mitotic cortical actin assembly, a key driver of mitotic rounding, is modulated during mitosis. Quantification of cortical F-actin accumulation in mosaic LifeAct-GFP expressing tECs revealed that both elongated motile tECs with high AR values (Fig. 6A-D; movie S12) and less-protrusive tECs with low fAR values (Fig. 6F-I; movie S13) efficiently accumulated mitotic cortical F-actin at the widest point of the cell body. In contrast, upon prophase entry, mitotic cortical F-actin only accumulated at detectable levels at leading membrane processes of less-protrusive tECs and was absent from elongated membrane processes of high AR tECs that failed to retract in isomorphic division (Fig. 6A-D,F-I). Likewise, isomorphic mitotic human ECs in 3D assays also failed to recruit normal levels of cortical F-actin to elongated protrusions (fig. S9A-D), consistent with the impact of cell length on the ease of mitotic rounding (Fig. 3A-C). Analysis of the total time spent in mitosis indicated that ease of mitotic rounding (as judged by the speed of membrane protrusion retraction) was the key driver of extent of mitotic rounding in vivo, rather than simply the total time spent rounding (fig. S10A,B). Indeed, both short and elongated tECs spent equivalent time in mitosis and had the same time available to round (fig. S10C). Consistent with higher ease of rounding, less-protrusive tECs that robustly assembled cortical actomyosin fully disassembled their filopodial protrusions and rapidly retracted membrane processes upon mitotic entry (Fig. 6J,K; movie S13). In contrast, isomorphic tECs retained active filopodia in mitosis, failed to retract their membrane processes, and evaded full mitotic rounding (Fig. 6E,K; movie S12). Thus, efficiency of mitotic rounding is markedly reduced in elongated tECs in vivo.

To also assess how myosin II activity is impacted by isomorphic division we monitored the mitotic spatial distribution of wild-type myosin-light-chain (MRLC), a rate-limiting component of the non-muscle myosin II complex and key determinant of mitotic cortical contractility (28, 29, 36) (Fig. 6L). Whilst mosaic expression of MRLC did not impact interphase tEC elongation, we observed disruption of isomorphic divisions, with even highly protrusive tECs undergoing rapid membrane retraction and extensive rounding to generate near-symmetric sized daughters (Fig. 6L-O; fig. S10D-H movie S14). Thus, even subtle manipulation of the levels of a single actomyosin modulator can switch tECs from an asymmetric to symmetric division outcome.

Likewise, treatment of embryos with the phosphatase inhibitor calyculin-A (CalyA), which inhibits MRLC phosphatase and enhances mitotic cortical contractility (28), perturbed isomorphic division, drove ectopic mitotic cell rounding, and triggerred a switch to more symmetric cell divisions (Fig. 6L-O; fig. S10D-I; movie S15). Following both manipulations, tEC division was now indistinguishable from less-protrusive control cells that rounded in mitosis, and significantly different to protrusive control cells that exhibited isomorphic division (Fig. 6M-O; fig. S10H). Modulation of mitotic rounding by CalyA enabled assessment of the impact of isomorphic division on post-mitotic asymmetry in Vegfr activity. In control embryos, asymmetric divisions generated daughters with asymmetry in Vegfr-mediated pErk (Fig. 6P), as expected (4, 6, 7). In contrast, perturbation of mitotic rounding significantly reduced post-mitotic Vegfr asymmetry, prevented creation of extreme differences in Vegfr activity (pErk ratios ≥4), and randomised daughter pErk levels such that asymmetry was often inverted (Fig. 6P). Thus, loss of pErk asymmetry is sufficient to perturb post-mitotic specification of daughters with distinct tip-stalk leader-follower migratory behaviour, perturbing zebrafish intersegmental vessel elongation (Fig. S10J). A switch to isomorphic division and tuning of the extent of mitotic rounding can thus induce post mitotic asymmetry in EC signalling state in vivo.

## Rab4 endosomes couple interphase cell morphology to fate determinant partitioning

**Fig 7:**
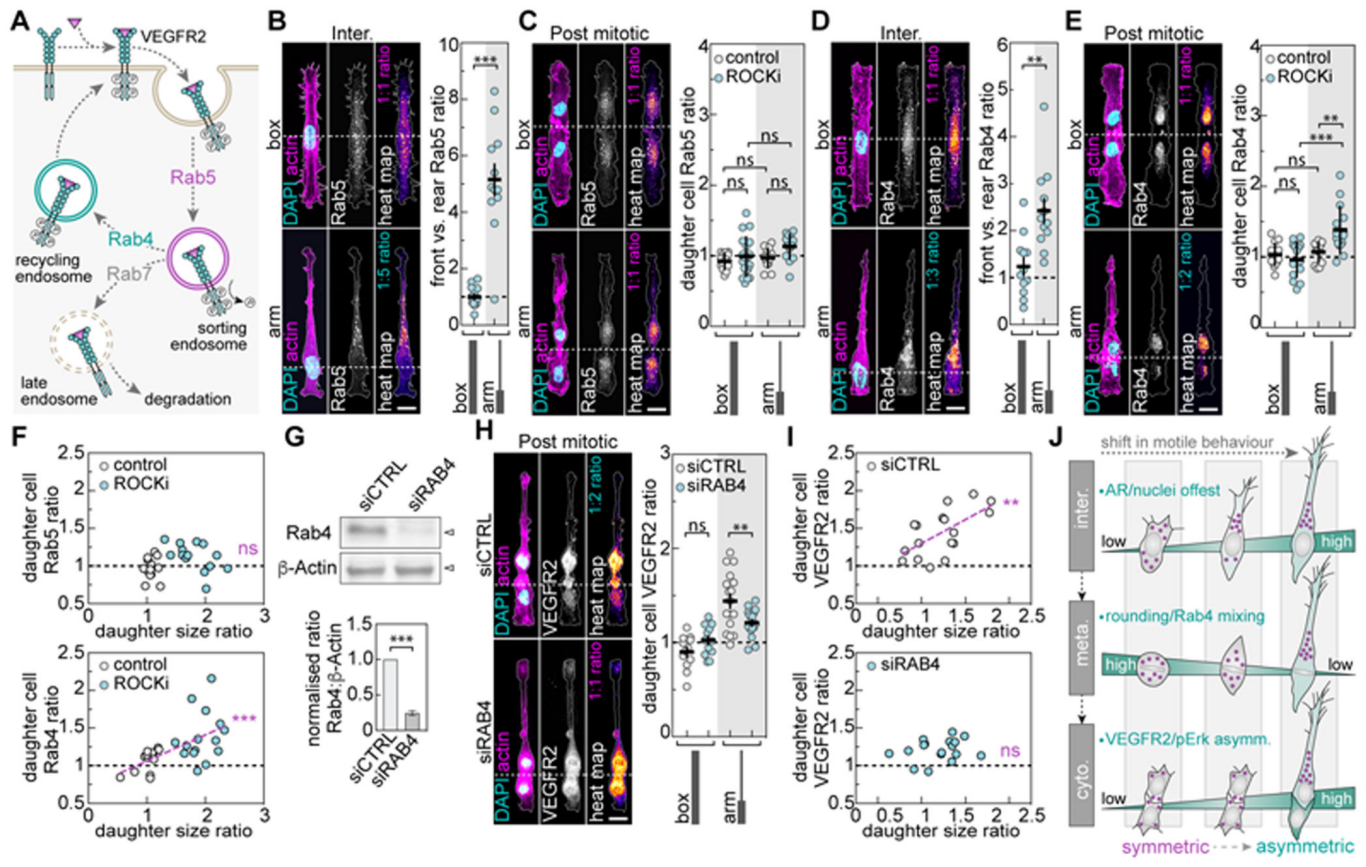
Rab4 endosomes drive asymmetric VEGFR2 partitioning in isomorphic division. (**A**) Rab GTPases and VEGFR2 trafficking. (**B,D**) Interphase ECs immunostained for Rab5 (B) or Rab4 (D; line indicates nuclei position). Plot of the ratio of Rab5 (B) or Rab4 (D) fluorescence intensity forward versus rearward of nuclei (*n*=13 Rab5 box, *n*=12 Rab5 arm; *n*=12 Rab4 box, *n*=12 Rab4 arm). (**C,E**) Post-mitotic ECs seeded on box/arm shapes in the presence ROCKi and immunostained for Rab5 (C) or Rab4 (E, line indicates cell-cell boundary). Plot of Rab5 (C) or Rab4 (E) daughter fluorescence intensity ratio on box/arm shapes in the absence or presence ROCKi (*n*=13 Rab5 box control, *n*=19 Rab5 box ROCKi, *n*=14 Rab5 arm control; *n*=15 Rab5 arm ROCKi; *n*=16 Rab4 box control, *n*=14 Rab4 box ROCKi, *n*=13 Rab4 arm control; *n*=15 Rab4 arm ROCKi). (**F**) Daughter size ratio versus the daughter ratio of either Rab5 or Rab4 fluorescence intensity for control and ROCKi-treated ECs seeded on arm shapes (black lines indicate symmetry; magenta line indicates Rab4 asymmetry; sample sizes as in panels C and D). (**G**) Immunoblot for Rab4 and β-Actin in control (siCTRL) or *RAB4A-* and *RAB4B*-targeting (siRAB4) siRNA-treated ECs and densitometric analysis (*n*=3). (**H**) siCTRL or siRAB4-treated ECs on arm shapes in the presence ROCKi fixed after division and immunostained for VEGFR2 (line indicates cell-cell boundary). Plot of the ratio of daughter VEGFR2 fluorescence intensity (*n*=12 siCTRL box, *n*=12 siRAB4 box, *n*=17 siCTRL arm, *n*=17 siRAB4 arm). (**I**) Daughter size ratio versus daughter VEGFR2 fluorescence intensity ratio for siCTRL- or siRAB4-treated ECs seeded on arm-shapes in the presence of ROCKi (black lines indicate symmetry; magenta line indicates VEGFR2 asymmetry; sample sizes as in panel H). (**J**) Relationship between isomorphic division, Rab4 endosome mixing and VEGFR2 inheritance. Data are mean ± s.d. ***P=<0.0010, **P=<0.0085, ns *P*=>0.0899. Two-tailed unpaired t-test for B,D,G and H. Unpaired ANOVA and Tukey’s multiple comparison test for C and E. Two-tailed Pearson’s correlation coefficient for F (*r*=0.5885) and I (*r*=0.6322). (B,C,D,E,G and H). For B,C,D,E, and H, scale bars, 10µm.

Whilst these observations confirm that interphase cell morphology tunes the mode, symmetry, and outcome of mitosis, it was unclear how cells sense and record prior shifts in pre-mitotic geometry to segregate mitotic fate determinant accordingly. However, the notably vesicular distribution of VEGFR-2 in interphase and mitosis indicated potential involvement of endosomal compartment dynamics (Fig. 5A,B; fig. S8A-D). In interphase, VEGFR-2 is predominantly distributed in Rab5-positive sorting endosomes, Rab4-positive recycling endosomes and Rab7-positive late endosomes (37, 38) (Fig. 7A). Considering that Rab7 targets VEGFR-2 for lysosomal degradation and is thus unlikely to participate in asymmetric mitotic partitioning, we assessed the spatial dynamics of Rab5-positive and Rab4-positive endosomal compartments in interphase and mitosis. Shifting ECs from symmetric to asymmetric micropatterns induced a notable front-rear polarisation of the Rab5-positive sorting endosomes in interphase (Fig. 7B). However, despite this extreme pre-polarisation, Rab5-positive endosomes were efficiently mixed and symmetrically segregated in mitosis, even in asymmetric isomorphic divisions that generated daughters of distinct size (Fig. 7C). Thus, mechanisms exist to overcome interphase asymmetry and ensure equal partitioning of this key cellular component, likely via known interaction of Rab5 with the mitotic spindle (39). In contrast, Rab4-positive recycling endosomes exhibited much weaker front-rear polarisation in interphase cells (Fig. 7D), like observed with VEGFR-2 (Fig. 5A). However, unlike Rab5, the Rab4-positive compartment was not mixed during isomorphic division, and this pre-polarization was maintained throughout mitosis leading to significant asymmetric partitioning of the Rab4-positive endosomes (Fig. 7E). Consequently, differential inheritance of Rab4, but not Rab5, was tightly associated with daughter cell size asymmetry (Fig. 7F), suggesting a causal role in asymmetric segregation of VEGFR-2. To test this idea, we silenced *RAB4A* and *RAB4B* using siRNAs (Fig. 7G) and determined the impact on VEGFR-2 mitotic partitioning. In the absence of Rab4, VEGFR-2 distribution remained highly vesicular and pre-polarised in asymmetric interphase cells, consistent with rerouting to Rab5-positive sorting endosomes (40). However, asymmetric mitotic partitioning of VEGFR-2 in isomorphic divisions was significantly perturbed in the absence of Rab4, generating post-mitotic cells that inherited near-equal amounts of VEGFR-2 despite clear asymmetry in daughter size (Fig. 7H). Indeed, normal tight coupling between daughter cell size ratio and asymmetry in post-mitotic inheritance of VEGFR-2 was entirely lost upon *RAB4* silencing (Fig. 7I). Thus, Rab4-positive recycling endosomes can record features of interphase morphology and thereby couple shifts in morphogenetic behaviour to the differential partitioning of fate determinants during division.

## Discussion

Here we have shown that switches in morphogenetic behaviour that induce shifts in mesenchymal-like cell morphology profoundly alter the mode of cell division and ultimately trigger key transitions in cell state in vivo. Thus, cell morphological dynamics exert the structural change that defines tissue form and concomitantly instruct shifts in the mode, symmetry, and outcome of mitosis that spatiotemporally remodels cell state and tissue diversity (Fig. 7J). These observations also uncover dynamic modulation of the ‘extent’ of mitotic rounding in isomorphic divisions as a trigger for asymmetric division and cell heterogeneity. Furthermore, we identify the conserved suite of interphase geometric rules that couple interphase shape to this modulation of division symmetry and outcome. Finally, we identify how cells achieve this mechanistic coupling, utilising specific endosomal compartments to sense and record shifts in interphase morphology and partition mitotic fate determinant accordingly. Considering that this framework can be exploited in vitro to force predictable post-mitotic asymmetry of any mesenchymal-like cell type tested to date, cues encoded in interphase morphology are likely a conserved trigger for asymmetric division and cellular heterogeneity in many tissue contexts. Indeed, there are clear benefits to using interphase morphology to instruct changes to daughter identity. For example, differential fate decisions can be made highly responsive to rapid changes in mesenchymal-like cell behaviour, with even subtle shifts in morphogenetic movements triggering very distinct mitotic outcomes. Moreover, maintenance of interphase morphology in isomorphic division enables extensive information on pre-mitotic cell state to be passed on to daughters, most of which is lost and reset in classical mitotic rounding. Indeed, isomorphic division is a much simpler means to retain information on interphase polarity than the more specialised and complex mechanisms that need to be employed by asymmetrically dividing cells that round in mitosis (3).

These data also raise fundamental questions about our current understanding of the mechanisms of division. For example, if division is frequently isomorphic, does the polarized interphase distribution of other key organelles influence their post-mitotic partitioning? Likewise, our work suggests complex roles for interphase cell adhesion in the control of mitosis. In contrast to stem cells (41–43), asymmetric interphase adhesion does not induce asymmetric division of ECs that undergo mitotic rounding. However, shifts in pre-mitotic environment and adhesion status do appear to trigger the switch to isomorphic division. Precisely how interphase cell adhesion influences mitotic symmetry thus remains unclear. Furthermore, mitotic rounding has long been considered critical for robust spindle assembly, chromosome capture and high-fidelity division (13, 14, 16). Understanding the mechanisms by which isomorphic cells evade the chromosome mis-segregation defects and mitotic checkpoints normally triggered by non-round divisions will be of great interest.

## Materials and Methods

### Zebrafish and treatment

Embryos and adults were maintained under standard laboratory conditions as described previously (44) and experiments were approved by the University of Manchester Ethical Review Board and performed according to UK Home Office regulations. For experiments, embryos were not selected for gender and were used between 15 and 32 hours post fertilization (hpf), unless otherwise stated. *Tg(kdrl:rasCherry)*^*s896*^, *Tg(kdrl:EGFP)*^*s843*^, *Tg(kdrl:nlsEGFP)*^*zf109*^, *Tg(clbnb:lyn GFP)* and *Tg(-4.9sox10:Hsa.HIST1H2BJ-mCherry-2A-GLYPI-EGFP)* strains were established previously (8, 45–47). *Tg*(*kdrl:Cerulean-H2B*) and *Tg*(*kdrl:2xlynGFPnls*) were generated using Tol2 transposon transgenesis. The pTol2 kdrl:Cerulean-H2B and pTol2 kdrl:2xlynGFPnls constructs were created by inserting Cer-H2B or GFPnls into pTol kdrl:GFP (46) or kdrl:2xlynRFP (10), respectively, after removing GFP or RFP. To achieve mosaic expression, 32 pg of pTol2 kdrl:lifeact-EGFP (48), pTol2 fli:GFP-α-tubulin (4) or pTol2 fli1:GFP-MRLC plasmid were co-injected with 32 pg Tol2 mRNA into one-cell stage *Tg(kdrl:rasCherry)*^*s896*^ zebrafish embryos. The pTol2 fli1:GFP-MRLC plasmid was created by inserting GFP-MRLC2 (49) (a gift from the laboratory of R. Das) into pTol2Dest (50) behind the basfli1ep promoter (51) using the Gateway system. For treatment with calyculin A (Abcam ab141784), embryos were incubated in 100-200 nM drug for 2 h prior to and throughout imaging.

### Mouse and treatment

In this study, we used the following mouse strains: R26-mTmG (52) and Cdh5CreERT2 (53). Mice were maintained and bred at the Instituto de Medicina Molecular João Lobo Antunes (IMM-JLA) under standard husbandry conditions and under Portuguese regulations. To induce recombination and mGFP expression, 4-hydroxytamoxifen (Sigma-Aldrich, H6278) was injected intraperitoneally (0,2 μg/g) at post-natal day 3 and eyes were collected at P6. Both males and females were used, without distinction. Animal experimentation was carried out in compliance with EU Directive 86/609/EEC and recommendation 2007/526/EC regarding the protection of animals used for experimental and other scientific purposes. Animal procedures were performed under supervision by the IMM-JLA Animal Ethics Committee (ORBEA), under the project licenses AWB_2015_10_CF_Polaridade and AWB_2021_02_CF_Vascular, and approved by the Portuguese Animal Ethics Committee regulatory body (DGAV), project licenses 0421/000/000/2016 and 0421/000/000/2021.

### Cell culture and treatment

All cells were maintained at 37°C, 5% CO2, with regular passages. Primary human umbilical vein endothelial cells (HUVECs) were cultured on 0.1% gelatin-coated dishes (Millipore) in Endothelial Growth Medium 2 (EGM-2) with the addition of a supplement pack (both Promocell) and 50mg/ml gentamycin (Sigma). Cells were detached using Trypsin-EDTA (Sigma). Primary mouse neural progenitor cells (NPCs) were isolated from mouse E12.5 forebrain and cultured in complete NS media as previously described (54), supplemented with 10ng/ml FGF-2 (PeproTech, Cat# 100-18B), 10ng/ml EGF (PeproTech, Cat# 100-18B) and 2μg/ml laminin (Sigma, Cat# L2020). Primary human pulmonary fibroblasts (HPF-Cs) were cultured on 0.1% gelatin-coated dishes in Dulbecco’s Modified Eagle’s Medium (DMEM; Sigma D5796) with 10% foetal calf serum. HPF-Cs used in 3D fibrin gel bead assays were moved to complete EGM-2 the day before use. For lentiviral work, HUVECs were transduced with lentiviruses containing GFP-actin biosensor (LentiBrite, Sigma 17-10204) at a multiplicity of infection (MOI) of 40 and incubated overnight. Cells were kept for 48 h before positive cells were selected by fluorescence activated cell sorting. For HUVEC 2.5D co-culture assays, HPF-Cs were first seeded in 35mm cell culture dish and maintained until confluent in DMEM for at least 72 h. During the last 24 h of growth, media was changed for EGM-2. Then, HUVECs expressing GFP-actin following lentiviral infection were seeded on top of the confluent fibroblasts and cultured for another 72 h before live-imaging. HUVEC 3D fibrin gel bead assays were performed as previously described (55). Once sprouts had formed (but before lumen formation) the media was changed 2 h before fixation to boost cell division. HPF-C feeder cells were then removed using 10x trypsin for 30 min at 37ºC and fibrin gels fixed for staining using 4% PFA for 30 mins. For drug treatments, prior to live imaging cells were incubated in fresh media supplemented with 100 μM (NPCs) or 200 μM (HUVECs) Y-27632 (Sigma), or with 50 μM Blebbistatin (Sigma) and 20mM HEPES (Gibco). Gene knockdown was achieved using AllStars negative control siRNA (Qiagen 1027280) or gene-specific FlexiTube siRNAs (Qiagen HsRab4A_6 – SI02655030, HsRab4B_6 – SI02662793). 0.3μM siRNA was diluted in 200μl Opti-MEM containing 1.5% v/v GeneFECTOR (Venn Nova). This mix was then added to a well of a 6-well plate containing 1ml Opti-MEM and the sample was incubated at 37°C for 3 h. Opti-MEM was then replaced with endothelial growth media and samples were allowed to recover for 48-72 h.

### Micropatterning

Micropatterning was performed using the PRIMO method (Alvéole) as previously described (26) with the following adaptations. All template patterns were designed using FIJI (ImageJ). Glass bottom dishes (35mm with 20mm center; Avantor) were used along with Polydimethylsiloxane (PDMS) stencils (Alvéole). Once incubated with Poly-L-Lysine-Poly-ethylene glycol (PLL-PEG) and photo-initiator PLPP (Alvéole) the dishes were patterned using a Nikon HE Widefield microscope combined with the Primo device and Leonardo software (Alvéole). Patterns were then coated with Fibronectin or Fibronectin-Alexa Fluor 488 (10µg/ml; Invitrogen) diluted in PBS for experiments with HUVEC’s, or with Laminin (10µg/ml; Sigma) diluted in PBS for experiments with NPC’s. HUVECs or NPCs were seeded onto Fibronectin/Laminin coated micropatterns (approx. 70,000 cells per dish) in either complete EGM2 or NPC media (supplemented with 10ng/ml EGF and 10ng/ml FGF2), as appropriate. Cells were incubated for 2-3 h and then the cell media was replaced with fresh media further supplemented with DMSO, Y-27632 or Blebbistatin prior to live imaging. For assessment of VEGFR-2-mediated pERK activity, cells were seeded onto micropatterns in minimal serum free media (OPTI-MEM). After 2 h, when cells were settled, media was exchanged for EGM2 supplemented with 200µM Y-27632 and 50ng/ml VEGF-165.

### Live imaging

Movies of zebrafish endothelial cells were acquired as previously described (4). Briefly, live embryos were mounted in 1% low-melting agarose (containing 0.1% tricaine) in glass-bottom dishes and were continually perfused with embryo water supplemented with 0.0045% 1-phenyl-2-thiourea and 0.1% tricaine at 28 °C using an in-line solution heater and heated stage controlled by a dual-channel heater controller (Warner Instruments). Embryos were imaged using 20× - or 40× -dipping objectives on either a Zeiss LSM 700 or 980 Airyscan confocal microscope and Z-stacks acquired every 1 to 3 min, unless otherwise indicated. Movies of zebrafish trunk neural crest and posterior lateral line primordium were acquired as previously described (8, 47).

Movies of HUVECs in 2.5D co-culture assays were acquired overnight at 37°C using a Nikon HE WideField microscope with a 20× objective. Images were acquired every 5 min. During acquisition, cells were continually maintained at 37°C and 5% CO_2_ in complete EGM-2 media (Promocell) complemented with 20mM HEPES buffer (Gibco). Movies of HUVECs and NPCs on micropatterns were acquired on an Eclipse Ti inverted microscope (Nikon) using a 10x/0.45 SPlan Fluor objective, the Nikon filter set for Brightfield and a pE-300 LED (CoolLED) fluorescent light source. The imaging software used was NIS Elements AR.46.00.0. Point visiting was used to allow multiple positions to be imaged within the same time-course and cells were continually maintained at 37°C and 5% CO_2_. Images were collected using a Retiga R6 (Q-Imaging) camera with a single z-optical plane being captured every 5 min for 2-18 h, as appropriate.

Movies of mouse melanoblasts were acquired as previously described (56).

### Antibody labelling

For HUVEC 3D fibrin gel bead assays, fixed fibrin gels were mechanically loosened from the dish to aid antibody penetration. Gels were then permeabilized (0.5% triton for 1 h) and blocked (5% goat serum, 2% BSA and 0.2% tween for 3 h). Followed by staining with primary antibodies diluted 1:200 (β-actin (Cell signaling 4967), Tubulin (Cell Signaling 2128) and phospho-Myosin Regulatory Light Chain (Cell Signaling 3671S)) for ~40 h at 4 °C and secondary antibodies diluted 1:500 (Invitrogen Alexa fluor: goat anti-rabbit 488, A11008 and goat anti-rabbit 568, A11011) for 4 h at room temperature or overnight at 4 °C. Gels were then removed from the plate and placed bottom side up in a glass bottom culture dish (MatTek Corporation P50G-1.5-14-F), mounting media (Vectashield with DAPI, Vector laboratories) was added, a coverslip placed on top and sealed with nail varnish prior to imaging. For micropatterned HUVECs and NPCs, cells were live imaged for 2-5 h to capture divisions before media was washed and cells quickly fixed in 4% PFA (Sigma), then permeabilized in 70% ethanol. Cells were incubated with primary antibodies in PBS for 1 h at room temperature (anti-VEGFR2 (R&D Systems MAB3572), anti-Dll4 (Invitrogen PA5-85931), anti-Dll1 (Abcam 10554), anti-Rab4 (Invitrogen PA3-912), anti-Rab5 (Cell Signalling C8B1), anti-pERK (Cell Signalling #4370)). Followed by secondary antibodies (Invitrogen Alexa fluor: goat anti-mouse 488, A32723, goat anti-rabbit 488, A11008, and goat anti-rabbit 647, A21246), DAPI (1/10,000) and 1:40 Rhodamine-Phalloidin (Invitrogen) in PBS at room temperature for 20 min. Finally, stained cells were mounted with ProLong Gold Antifade Mountant (Thermo Fisher Scientific). Images of all stained 2D/2.5D HUVECs and NPCs were acquired either on a Zeiss LSM 980 Airyscan confocal microscope, using a Zeiss Plan-Apochromat 63x/1.4 Oil lens and Z-stack spacing of 0.5µm, or on a Olympus IX83 inverted microscope using Lumencor LED excitation, a 60x/ 1.42 Plan Apo objective and the Sedat filter set (Chroma 89000). For the Olympus IX83, images were collected using a R6 (Qimaging) CCD camera with 15 slices acquired at a Z optical spacing of 0.2μm. HUVECs in the fibrin bead assay were imaged using a 40×-dipping objective on a Zeiss LSM 700 confocal microscope, with Z optical spacing of 1μm.

Staining of zebrafish embryos for pErk was performed as previously (4). Briefly, *Tg*(*kdrl:2xlynGFPnls*) embryos were live-imaged to capture mitosis and then flash fixed in ice-cold 4% PFA. Embryos were then washed in 100% MeOH, incubated with 3% H_2_0_2_ in MeOH on ice for 60 min and then washed again in 100% MeOH. Embryos were then incubated at -20°C for 2 days in MeOH, then equilibrated with PBT (PBS, 0.1% Tween-20) washes and cryoprotected in 30% sucrose in PBT at 4°C overnight. Following this, embryos were equilibrated in PBT, immersed in 150 mM Tris-HCl (pH 9.0) for 5 min and then heated to 70°C for 15 min. Two PBT washes and a wash in dH_2_O for 5 min was then followed by addition of ice-cold acetone for 20 min at -20°C. This was followed by PBT washes, one TBST (TBS, 0.1% Tween-20, 0.1% Triton X-100) wash and incubation overnight at 4°C with block solution (TBST, 1% BSA, 10% goat serum). Embryos were then incubated with anti-pERK antibody (1:250, Cell Signalling #4370) in blocking buffer overnight at 4°C. Next, embryos were washed in TBST at room temperature followed by a Maleic buffer wash (150 mM Maleic acid, 100 mM NaCl, 0.001% Tween-20, pH 7.4) for 30 min. Embryos were blocked in 2% blocking reagent (Roche) in Maleic buffer (3 h at room temperature) and then incubated with goat anti-rabbit IgG-HRP (1:1000) in 2% blocking reagent in Maleic buffer overnight at 4°C. Embryos were finally washed in Maleic buffer and then PBS at room temperature, incubated with 50 µl amplification diluent with 1 µl Tyramide-Cy3 (Perkin Elmer) for 3 h at room temperature in the dark and then washed over several days in TBST at room temperature.

Preparation, antibody staining and imaging of mouse retinal endothelial cells was performed as previously described (57, 58).

### Image analysis

All analysis and measurements were made using ImageJ, except 3D rendering analysis which was performed using Imaris software. For zebrafish cell divisions, movies were Z projected using their maximum intensity and drift removed using the 3D drift correct plugin, if required. Phases of mitosis were defined as (1) Interphase: The frame prior to nuclear envelope breakdown and/or onset of mitotic shape change (for example, retraction of filopodia); (2) Metaphase: The frame before anaphase onset; (3) Post-mitotic daughter cells: The first frame where 2 daughter cells are apparent (~1 to 6 min after anaphase onset). Morphometric measurements (fig. S1A) at each time point were acquired upon creation of a cell shape “mask” in ImageJ, that was processed through a python script to identify the cell’s longest axis (medial spine) and widest point along this medial axis. From this the length and width measurements were then made. “Front length” was measured from the widest point to the front tip and “rear length” from the widest point to the rear tip. Aspect ratios were then calculated by dividing the relevant length by the width. For example, front aspect ratio was calculated by measuring the front length divided by the cell width at each time point. “Offset” was measured by dividing the front length by the rear length. From Anaphase onwards “Offset” was the length of the new tip cell divided by the length of the new stalk cell. Cell area and circularity were calculated in ImageJ using the original cell mask. Daughter cell size ratios were determined upon manual recording of each daughter cell area in ImageJ and then calculating the ratio of area of the new tip/leader cell versus the new stalk/follower cell. Cell front retraction was measured by overlaying the interphase and metaphase “long axis” masks and measuring the distance between the two cell tips. Retraction speeds were calculated by measuring the distance the tip of the cell moves at each time point in mitosis and averaging this over time. Shape change occurring during cell migration and the rate of migration were characterized as the change in front aspect ratio every 3 min and the Y axis movement of the cell tip every 3 min, respectively. These were then plotted as a rolling average every 30 mins.

For HUVEC and NPC divisions, the phases of mitosis were standardized as (1) Interphase: 12 frames before cytokinesis; (2) Metaphase: 2 frames before cytokinesis; (3) Cytokinesis: First frame with two daughter cells evident; (4) Post-mitotic daughter cells: 6 frames after cytokinesis. In the 3D bead assay the morphology of the DNA was used to determine phase of mitosis. Aspect ratios were determined by dividing the total cell lengths by their widths. Front aspect ratios were determined by dividing the front length (middle of nucleus or furrow position to front tip) by the width. “Offset” was measured by dividing front length by rear length (middle of nucleus/furrow position to tip/rear). Cell area and circularity calculated by Imagej after manually drawing around the cell. The “front retraction” was measured as the distance between the starting point of the front tip in interphase and its position in metaphase. Daughter cell size ratio was determined upon manual recording of daughter cell area in ImageJ and the calculation of the ratio of area of the new front (top) cell versus the new rear (bottom) cell.

For quantification of fluorescence intensity, sum intensity projections of raw image files were used to calculate fluorescence intensity ratios of cells using ImageJ software. Using the polygon tool, cell outlines were drawn and the integrated density of fluorescence of cells and background were measured, along with the cell area.

A Python script using the scikit-image library was used to define the medial spine of the cell shape masks generated in ImageJ. This takes the form of a network of pixels through the cell and the corresponding width of the cell at each pixel is calculated. The script then walks through all branches in the spine to find the longest possible path. This path is then marked as the longest axis of the cell and a red dot is generated to mark the widest (from the previously calculated widths) point of the shape along this axis. This script is called CharacteriseShape and is available on GitHub at https://github.com/chris-revell/CharacteriseShape.

For quantification of cell motility in vivo, daughter cell motility after division was measured using ImageJ by measuring the distance between the centre of the daughter tip or stalk nucleus and the base of the vessel (where the sprout leaves the dorsal aorta) for each time point. Time 0 was set as the first frame when two daughter nuclei were apparent (when the nuclear envelope has reformed) and the distances were then normalised by subtracting their starting position from each timepoint. Cell velocity was then calculated as an average of the distance moved (µm) every frame (15 min) after division.

For analysis of centrosome dynamics in vivo, centrosome and nucleus positions were identified as a high concentration of GFP-α-tubulin where microtubule array emanates and from the void in GFP-α-tubulin signal (which is lost at NEB), respectively. Spindle poles/centrosomes were identified at intense concentrations of GFP-α-tubulin associated with the nucleus following centrosome duplication. Offset of the centre of the spindle poles (frame before NEB) or established spindle (first frame in which two sides of spindle appear to meet) from the cell midpoint was calculated by measuring the position of the midpoint between the spindle poles along the total length of cell and dividing the front length by the rear length.

3D rendering of cells in vivo and in vitro was performed using Imaris software. Z stack images were imported into Imaris and the Surfaces tool was used to generate a 3D rendering of the surface of each cell/pair of daughter cells (using phalloidin or lyn-mCherry to indicate cell outline in vitro and in vivo, respectively) and the nucleus (DAPI, in vitro and cerulean-H2B, in vivo). This was performed automatically for in vitro images with a constant thresholding value and manually (by drawing cell outline in each Z plane) for in vivo images and the volume of each cell was calculated. Daughter cell volume ratios were calculated by dividing the volume of the top daughter by the bottom daughter (in vitro) or the tip daughter by the stalk daughter (in vivo). The offset of nuclei centre from the cell centre was calculated by measuring the distance between the two volumetric centres (marked using Imaris) and this was then expressed as a percentage of the total cell length.

### Immunoblotting

For immunoblotting, whole protein was extracted using RIPA buffer (25 mM Tris–HCl pH 7.6, 150 mM NaCl, 1% NP-40, 1% sodium deoxycholate and 0.1% SDS) containing 1:100 Protease Inhibitor Cocktail. Protein concentration was quantified using Pierce BCA Protein Assay Kit (Thermo Fisher Scientific) before denaturation with Laemmli Buffer (250 mM Tris–HCl pH 6.8, 2% SDS, 10% glycerol, 0.0025% bromophenol blue, 2.5% β-mercaptoethanol) at 95°C for 5 min. Proteins were separated using 10% Mini-PROTEAN TGX precast protein gels (Bio-Rad). Trans-Blot Turbo transfer system (Bio-Rad) was used to transfer proteins to PVDF membranes using manufacturer’s guidelines. Membranes were then blocked for 1hr at room temperature in 2.5% BSA (Sigma) in TBS containing 0.1% Tween-20. Primary antibodies (anti-Rab4 (Invitrogen PA3-912), anti-β-actin (Cell Signalling Technology 4967)) were diluted in blocking buffer and incubated overnight at 4°C. Membranes were then washed with TBS containing 0.1% Tween-20 before incubation with secondary antibodies (Anti-Rabbit-HRP (Cell Signalling Technology 7074)) diluted in blocking buffer for 1hr at room temperature, followed by more washes. Pierce SuperSignal West Atto immunoblotting substrate (Thermo Fisher Scientific) was used to develop chemiluminescent signal, which was then detected digitally using a ChemiDoc MP Imager (Bio-Rad).

### Statistics

All statistical analyses were performed using GraphPad Prism software (v.9.4.1). No statistical methods were used to predetermine sample sizes. Normality testing (Shapiro-Wilk) was performed to determine if datasets were suitable for parametric or non-parametric statistical tests. Either a two-tailed unpaired Student’s t-test or a two-tailed Mann–Whitney test were used for the comparison of two normally distributed or non-normally distributed datasets, respectively. An ordinary one-way ANOVA with Tukey’s multiple comparisons test was used to compare more than two normally distributed datasets. To compare more than two non-normally distributed datasets, an unpaired Kruskal–Wallis test and Dunn multiple comparison test was used. Error bars and P values are reported in the figures and/or figure legends. All statistical tests were performed on datasets acquired from at least three independent experiments. For each analysis, measurements were taken from distinct samples.

## Supplementary Material

Supplementary Material

## Data Availability

Python code used to quantify cell shape metrics can be found at https://github.com/chris-revell/CharacteriseShape and are archived in Zenodo (https://doi.org/10.5281/zenodo.14680048) (59). All data are available in the main text or the supplementary materials.
