# Supplementary material for: Interphase cell morphology defines the mode, symmetry, and outcome of mitosis
Source: Science. Author manuscript; Available in PMC 2026 Jul 14. (PMC7619237; doi:10.1126/science.adu9628)
Supplement: Supplementary Material [file EMS215956-supplement-Supplementary_Materials.pdf]

Science
AAAS

Supplementary Materials for

Interphase cell morphology defines the mode, symmetry and outcome of mitosis

Holly E. Lovegrove[1*†], Georgia E. Hulmes[1†], Sabrina Ghadaouia[1,2‡], Christopher Revell[1,3,4‡,5], Marta Giralt-Pujol[1,6‡], Zain Alhashem[7], Andreia Pena[8], Damian D. Nogare[9], Ellen Appleton[1,4], Guilherme Costa[10], Richard L. Mort[11], Christoph Ballestrem[1], Gareth W. Jones[4], Cerys S. Manning[1], Ajay B. Chitnis[9], Claudio A. Franco[8,12], Claudia Linker[7], Katie Bentley[3‡,5,13], Shane P. Herbert[1*]

Corresponding authors: Shane P. Herbert, shane.herbert@manchester.ac.uk; Holly E. Lovegrove, holly.lovegrove@manchester.ac.uk

The PDF file includes:

Figs. S1 to S10

Other Supplementary Materials for this manuscript include the following:

Movies S1 to S15

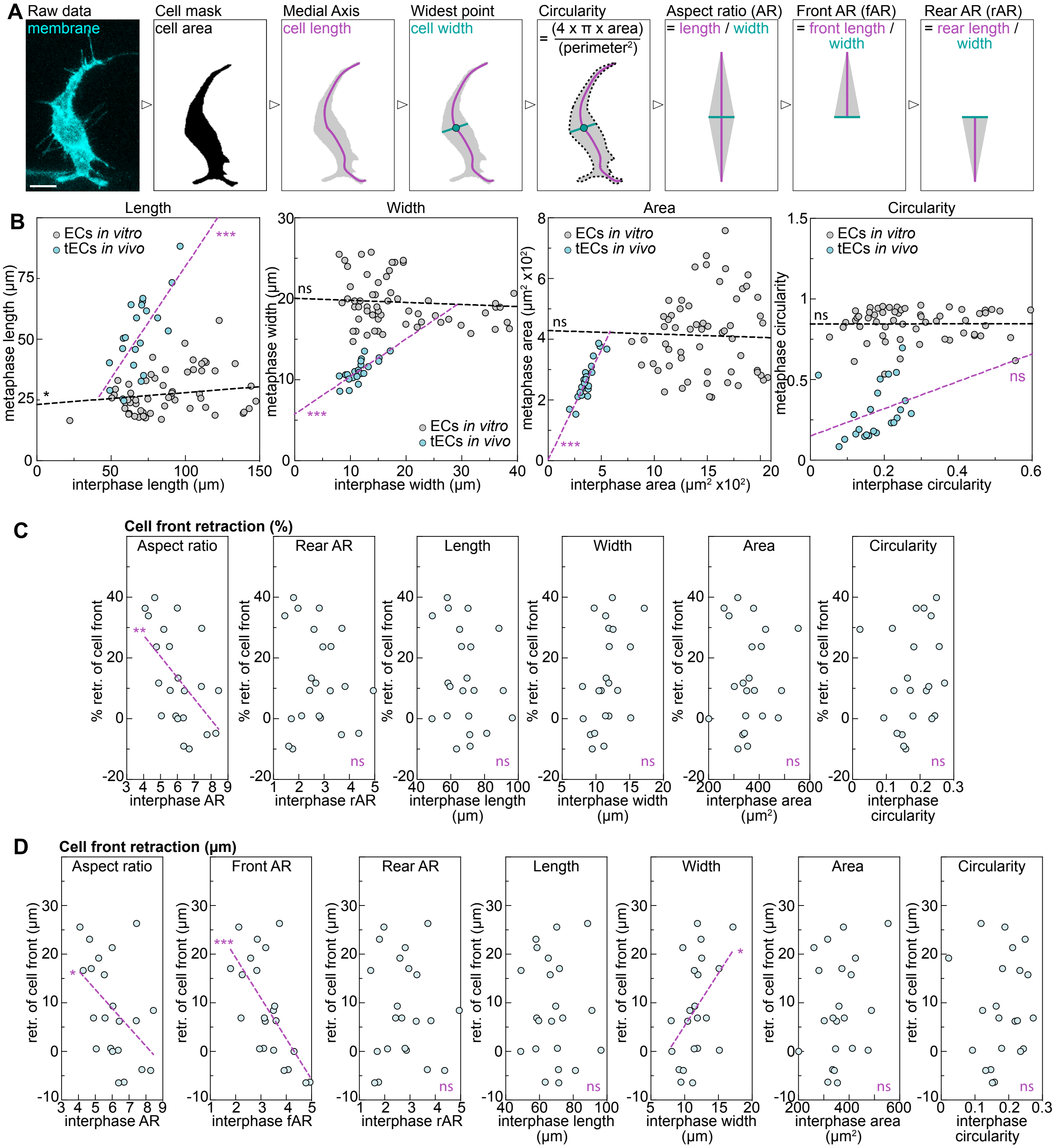
A
Raw data
membrane
Cell mask
cell area
Medial Axis
cell length
Widest point
cell width
Circularity
= (4 x π x area) / (perimeter²)
Aspect ratio (AR)
= length / width
Front AR (fAR)
= front length / width
Rear AR (rAR)
= rear length / width
B
Length
ECs in vitro
tECs in vivo
metaphase length (μm)
interphase length (μm)
Width
metaphase width (μm)
interphase width (μm)
Area
metaphase area (μm² x10²)
interphase area (μm² x10²)
Circularity
metaphase circularity
interphase circularity
C
Cell front retraction (%)
Aspect ratio
Rear AR
Length
Width
Area
Circularity
% retr. of cell front
interphase AR
interphase rAR
interphase length (μm)
interphase width (μm)
interphase area (μm²)
interphase circularity
D
Cell front retraction (μm)
Aspect ratio
Front AR
Rear AR
Length
Width
Area
Circularity
retr. of cell front (μm)
interphase AR
interphase fAR
interphase rAR
interphase length (μm)
interphase width (μm)
interphase area (μm²)
interphase circularity

Fig. S1. tEC cell morphology is associated with a switch to isomorphic division. (**A**) Schematic of the in silico morphometric image analysis pipeline used to extract cell morphological features. (**B**) Plots comparing cell length, width, area or circularity in interphase versus metaphase for tECs in vivo (magenta lines; *n*=22 cells) and ECs in vitro (black lines; *n*=60 cells; two-tailed Pearson's correlation coefficient; ****P*=<0.0003, **P*=0.0146, ns *P*=>0.2051; *r*=0.6958 length, *r*=0.7613 width, *r*=0.9021 area). (**C**) Plots comparing interphase tEC aspect ratio (AR), rear AR (rAR), length, width, area and circularity versus percentage retraction (retr.) of the cell front in mitosis (*n*=22 cells; two-tailed Pearson's correlation coefficient; ***P*=0.0095, ns *P*=>0.0559; *r*=-0.5399). (**D**) Plots comparing interphase tEC aspect ratio (AR), front AR (fAR), rear AR (rAR), length, width, area and circularity versus μm of retraction (retr.) of the cell front in mitosis (*n*=22 cells; two-tailed Pearson's correlation coefficient; ****P*=0.0009, **P*=<0.0318, ns *P*=>0.3183; *r*=-0.6553 fAR, *r*=-0.4591 AR). For A, scale bar, 10μm.

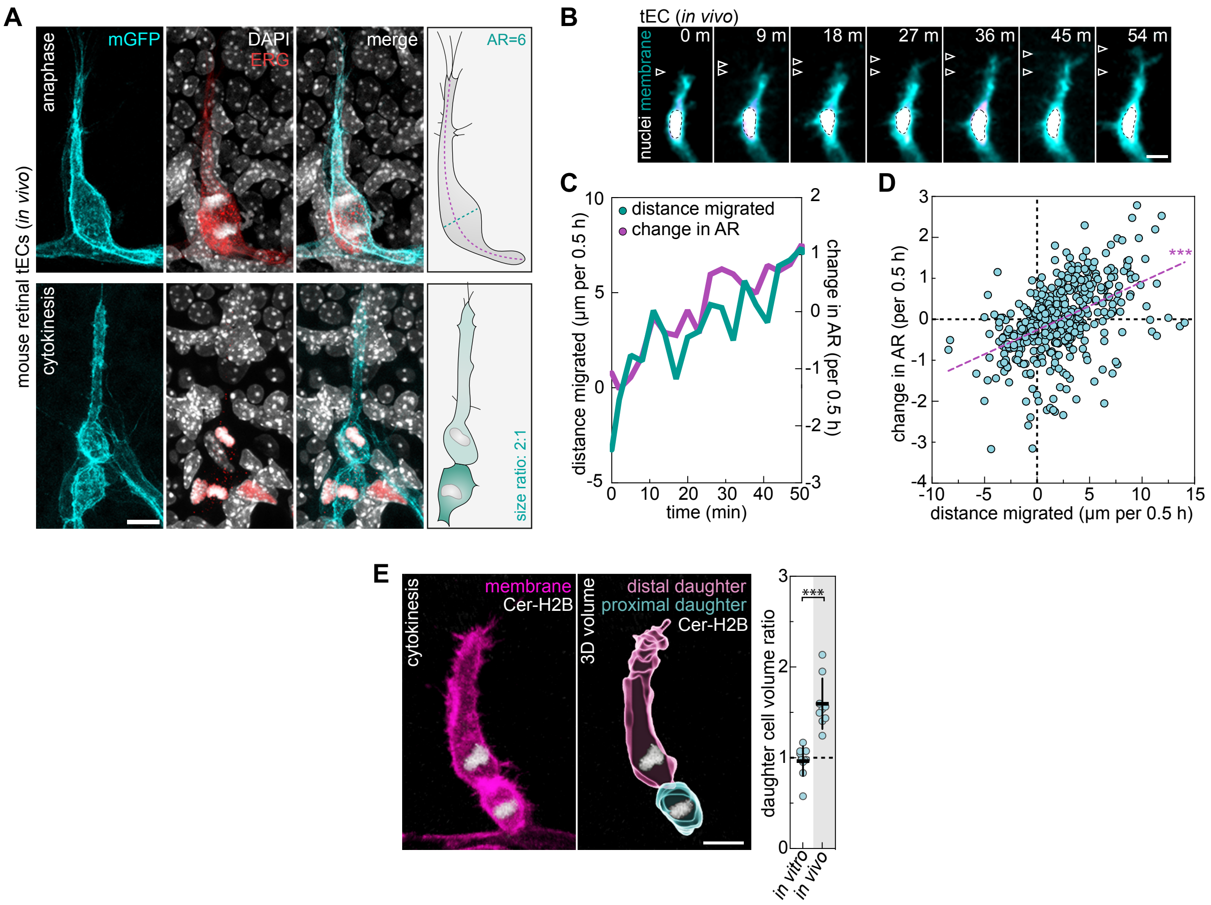

F

Cell type	Inter. AR	Meta. AR	n number	Reference.
Endothelial (tEC)	6.3	4.7	22	(see methods)
Trunk neural crest (tNC)	5.3	3.2	17	(see methods)
Posterior lateral line (PLLp)	2.1	1.1	14	(see methods)
Xanthophore (XP)	4.6	3.7	4	Walderich et. al. (2016) Nat. Commun., 7, 11462. doi: 10.1038/ncomms11462
Hypodermal (HD)	3.9	3.3	1	Budi et.al. (2011) PLoS Genet. 7(5) e1002044. doi: 10.1371/journal.pgen.1002044
Melanoblast (MB)	2.0	1.7	3	(see methods)
Primordial Germ (PGC)	1.3	1.0	2	Pfeiffer et.al. (2018) Dev. Biol. 436(2) 84-93. doi: 10.1016/j.ydbio.2018.02.014

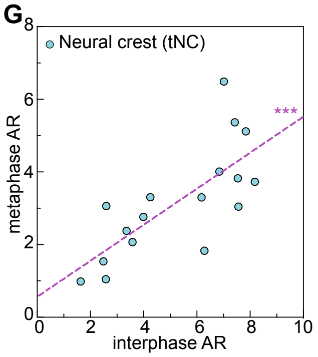

Fig. S2. The switch to isomorphic division is a conserved feature of motile mesenchymal-like cells. (**A**) Images of dividing mouse retinal tECs expressing mosaic mGFP and immunostained for the EC marker, ERG. Schematics show AR in anaphase and daughter size ratios following cytokinesis. (**B**) Still images of a migrating zebrafish tEC expressing 2xlyn-GFPnls and lyn-mCherry (arrowheads indicate the initial and end positions of the motile leading edge; black dashed line indicates nuclei). (**C**) Plot of both the change in aspect ratio (AR) and distance migrated over time for the tEC shown in panel A. (**D**) Plot comparing distance migrated versus change in AR (n=443 0.5 h time points taken from 6 tECs; two-tailed Pearson's correlation coefficient; ***P=<0.0001; r=0.4752). (**E**) Still images and 3D volume rendering of highly elongated post-mitotic lyn-mCherry-and Cerulean-H2B-expressing tEC daughters. Quantification of daughter cell volume ratio following division both for mitotic ECs in vitro and elongated mitotic tECs in vivo (n=10 in vitro cells; n=9 in vivo cells; two-tailed unpaired t-test, ***P=<0.0001). (**F**) Table showing AR in interphase and metaphase for the indicated motile cell types in vivo. (**G**) Plot comparing AR in interphase versus metaphase for tNC cells in zebrafish embryos (n=17 cells; two-tailed Pearson's correlation coefficient; ***P=0.0007; r=0.7395). Data are mean ± s.d. (E). For A, B and E, scale bars, 10μm.

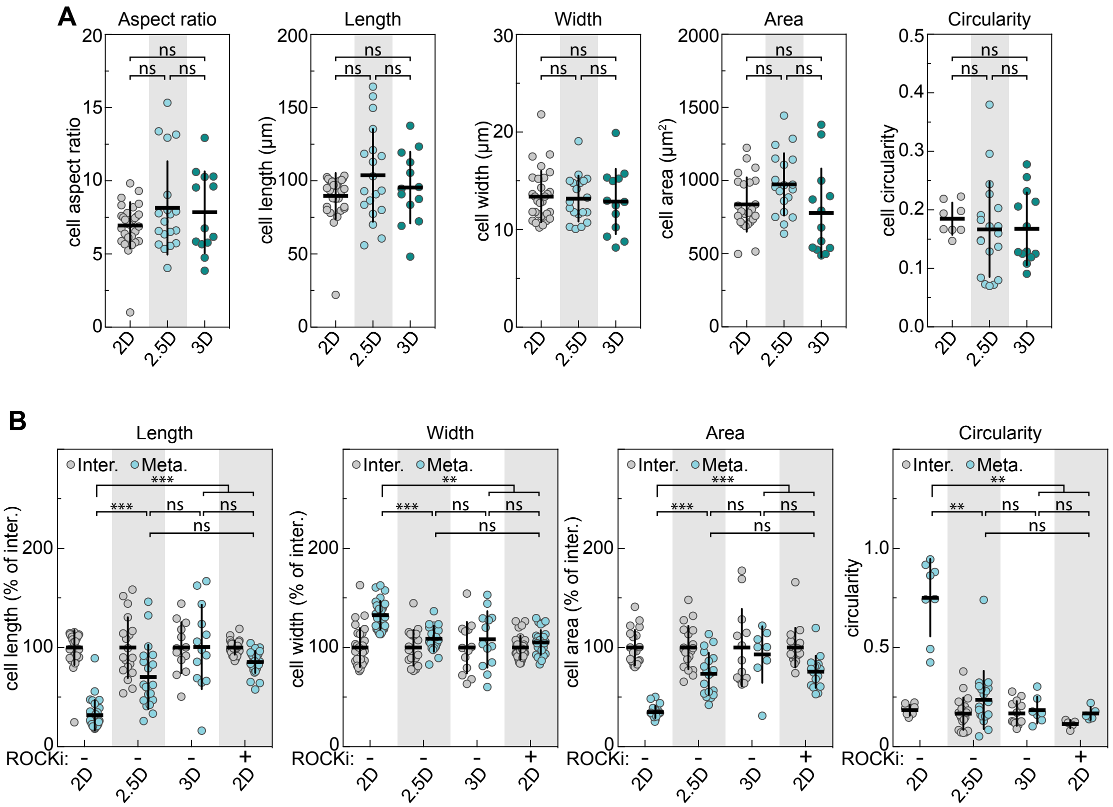

Fig. S3. Recapitulation of isomorphic division using 2D micropatterned cells. (**A**) Plots show quantification of either EC aspect ratio, length, width, area or circularity in interphase for ECs cultured in either 2D, 2.5D or 3D (*n*=21 2D cells, *n*=19 2.5D cells, *n*=13 3D cells; Unpaired Kruskal–Wallis test and Dunn multiple comparison test; ns *P*=>0.0541). (**B**) Plots show quantification of either EC length, width, area or circularity in interphase and metaphase for ECs cultured in either 2D, 2.5D, 3D or 2D in the presence of ROCKi (*n*=20 2D cells, *n*=19 2.5D cells, *n*=at least 8 3D cells, *n*=17 2D + ROCKi cells; Unpaired Kruskal–Wallis test and Dunn multiple comparison test; ****P*=<0.0003, ***P*=<0.0050, ns *P*=>0.2713).

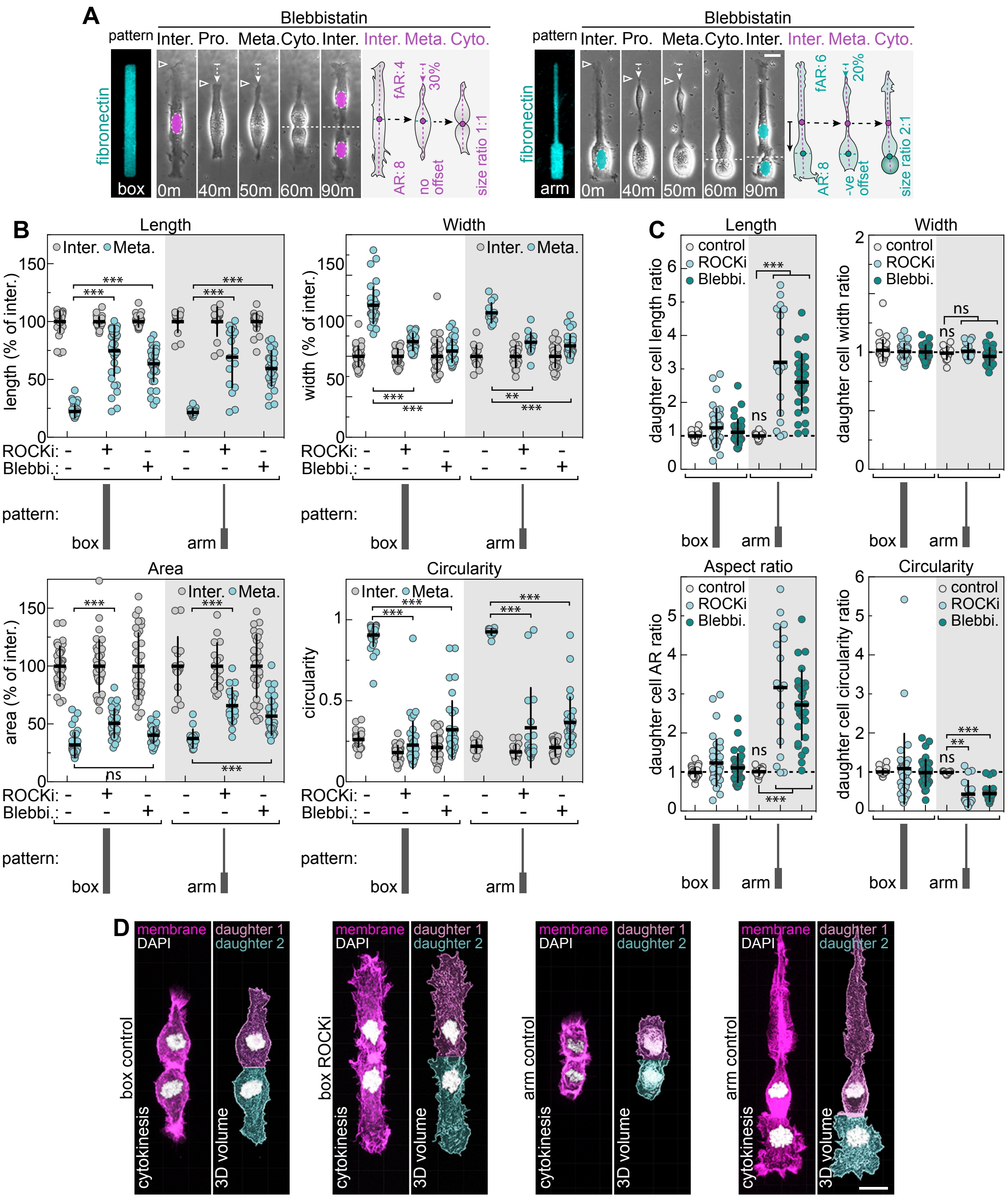
A
Blebbistatin
pattern
Inter. Pro. Meta. Cyto. Inter. Inter. Meta. Cyto.
fibronectin
box
0m 40m 50m 60m 90m
fAR: 4
30%
AR: 8
no offset
size ratio 1:1
arm
fAR: 6
20%
-ve offset
size ratio 2:1
B
Length
Width
Area
Circularity
Inter. Meta.
length (% of inter.)
width (% of inter.)
area (% of inter.)
circularity
ROCKi:
Blebbi.:
pattern:
box
arm
ns
C
Length
Width
Aspect ratio
Circularity
control
ROCKi
Blebbi.
daughter cell length ratio
daughter cell width ratio
daughter cell AR ratio
daughter cell circularity ratio
D
membrane
DAPI
daughter 1
daughter 2
box control
box ROCKi
arm control
cytokinesis
3D volume

Fig. S4. Interphase morphometric cues break the symmetry of isomorphic division. (**A**) Still images of human ECs seeded on micropatterned fibronectin shapes undergoing mitosis in the presence of Blebbistatin (arrowheads, dashed arrows and dashed lines indicate cell front, retraction of cell front and cell-cell boundary, respectively; nuclei are pseudocolored; Interphase (Inter.), Prophase (Pro.), Metaphase (Meta.), Cytokinesis (Cyto.)). Schematics show constant aspect ratio (AR), differing front AR (fAR), offset of the nuclei/metaphase plate/cleavage furrow (arrow), percentage retraction of the cell front and daughter size ratios. (**B**) Plots of either length, width, area or circularity in interphase and metaphase for ECs seeded on micropatterned fibronectin shapes in the presence or absence of ROCKi or blebbistatin (Blebbi.; *n*=36 control Box cells, *n*=36 ROCKi Box cells, *n*=27 Blebbi. Box cells, *n*=15 control Arm cells, *n*=17 ROCKi Arm cells, *n*=30 Blebbi. Arm cells; Unpaired Kruskal–Wallis test and Dunn multiple comparison test; ****P*=<0.0008, ***P*=0.0030, ns *P*=0.0605, all statistical comparisons are indicated by brackets). (**C**) Plots of post-mitotic daughter ratios in length, width, aspect ratio and circularity for ECs seeded on micropatterned fibronectin shapes in the presence or absence of ROCKi or blebbistatin (Blebbi.; *n*=36 control Box cells, *n*=36 ROCKi Box cells, *n*=34 Blebbi. Box cells, *n*=15 control Arm cells, *n*=17 ROCKi Arm cells, *n*=30 Blebbi. Arm cells; Unpaired Kruskal–Wallis test and Dunn multiple comparison test; ****P*=<0.0001, ***P*=<0.0010, ns *P*=>0.9999, versus control box, unless indicated by bracket). (**D**) Still images and 3D volume rendering of post-mitotic ECs seeded on micropatterned fibronectin box or arm shapes in the presence or absence of ROCKi (as quantified in Fig. 2G). Data are mean ± s.d. (B and C). For A and D, scale bars, 10µm.

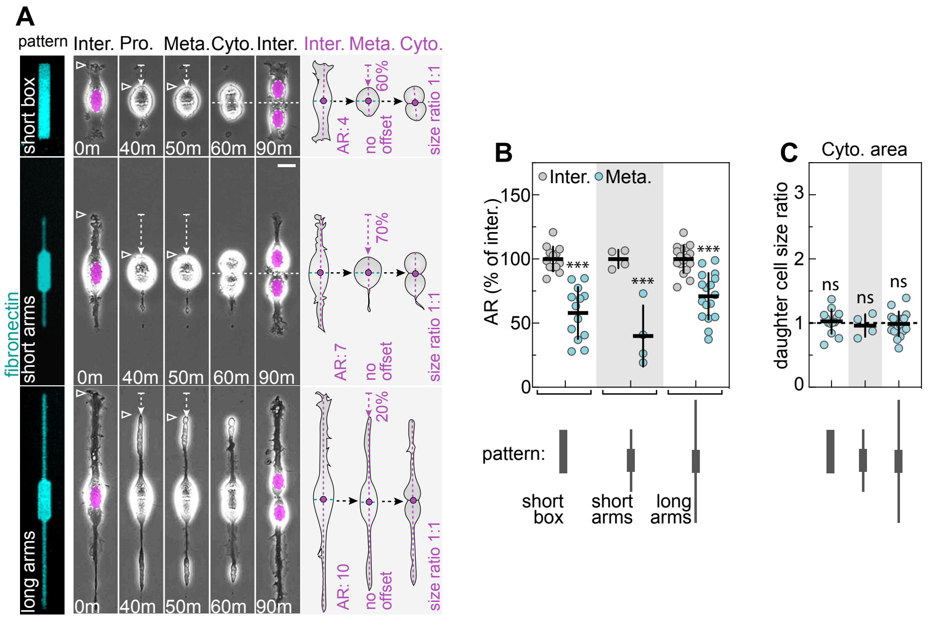

Fig. S5. Asymmetry in interphase morphology breaks the symmetry of isomorphic division. (**A**) Still images of human ECs seeded on micropatterned fibronectin shapes undergoing mitosis in the presence of ROCKi (arrrowheads, dashed arrows and dashed lines indicate cell front, retraction of cell front and cell-cell boundary, respectively; nuclei are pseudocolored). Schematics show differing aspect ratio (AR), no offset of the nuclei/metaphase plate/cleavage furrow, percentage retraction of the cell front and daughter size ratios. (**B**,**C**) Plots of either interphase and metaphase ARs (B) or of post-mitotic daughter size ratios (C) for ECs seeded on the indicated micropatterned fibronectin shapes in the presence of ROCKi (*n*=13 short box cells, *n*=4 short arm cells, *n*=17 long arms cells; Unpaired ANOVA and Tukey's multiple comparison test; ****P*=<0.0001, ns *P*=>0.9999, versus either interphase (B) or short box (C)). Data are mean ± s.d. (B and C). For A, scale bar, 10μm.

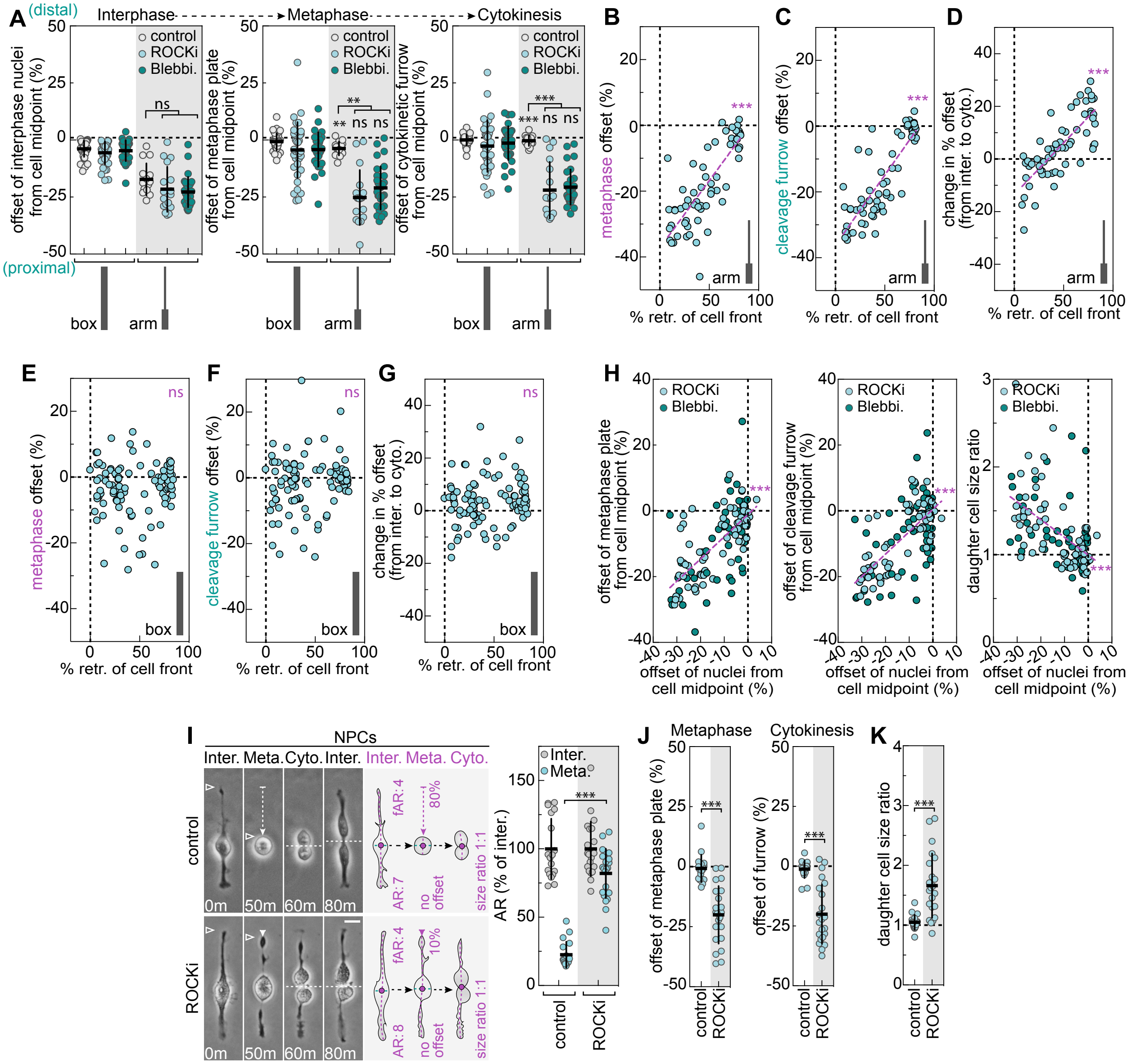
A
(distal)
(proximal)
Interphase
Metaphase
Cytokinesis
control
ROCKi
Blebbi.
offset of interphase nuclei from cell midpoint (%)
offset of metaphase plate from cell midpoint (%)
offset of cytokinetic furrow from cell midpoint (%)
ns
**
***
box
arm
B
C
D
metaphase offset (%)
cleavage furrow offset (%)
change in % offset (from inter. to cyto.)
% retr. of cell front
E
F
G
H
offset of metaphase plate from cell midpoint (%)
offset of cleavage furrow from cell midpoint (%)
daughter cell size ratio
offset of nuclei from cell midpoint (%)
I
NPCs
Inter. Meta. Cyto. Inter.
Inter. Meta. Cyto.
control
ROCKi
0m
50m
60m
80m
fAR: 4
AR: 7
80%
no offset
size ratio 1:1
AR: 8
10%
AR (% of inter.)
Inter.
Meta.
J
Metaphase
Cytokinesis
offset of metaphase plate (%)
offset of furrow (%)
K
daughter cell size ratio

Fig. S6. A switch to isomorphic division maintains an offset metaphase plate and cleavage furrow. (**A**) Plots of the offset of the nuceli, metaphase plate or cleavage furrow from the midpoint of cell length throughout division for ECs seeded on micropatterned fibronectin shapes in the presence or absence of ROCKi or Blebbi. (n=36 control box cells, n=36 ROCKi box cells, n=33 Blebbi. Box cells, n=15 control arm cells, n=17 ROCKi arm cells, n=30 Blebbi. Arm cells; Unpaired Kruskal–Wallis test and Dunn multiple comparison test; ***P=<0.0002, **P=<0.0031, ns P=>0.8264, versus interphase nuclei offset, unless indicated by bracket). (**B**,**C**,**D**) Plots comparing the percentage retraction of the cell front versus either offset of the metaphase plate from the EC midpoint (B; r=0.7981), the offset of the cleavage furrow from the EC midpoint (C; r=0.8360) or the change in offset occurring from interphase to cytokinesis (offset of the interphase nuclei versus offset of the cytokinetic furrow; D; r=0.7839) for ECs seeded on arm micropatterned fibronectin shapes in the presence or absence of ROCKi or blebbistatin (n=62 cells; two-tailed Pearson's correlation coefficient; ***P=<0.0001). (**E**,**F**,**G**) Plots comparing the percentage retraction of the cell front versus either the offset of the metaphase plate from the tEC midpoint (E), the offset of the cleavage furrow from the tEC midpoint (F) or the change in offset occurring from interphase to cytokinesis (offset of the interphase nuclei versus offset of the cytokinetic furrow; G) for ECs seeded on box micropatterned fibronectin shapes in the presence or absence of ROCKi or blebbistatin (n=106 cells; two-tailed Pearson's correlation coefficient; ns P=>0.0781). (**H**) Plots comparing offset of the interphase nuceli versus either offset of the metaphase plate, offset of the cleavage furrow or daughter cell size ratio division for ECs seeded on micropatterned fibronectin shapes in the presence of ROCKi or Blebbi (n=53 ROCKi cells, n=64 Blebbi. Cells; two-tailed Pearson's correlation coefficient; ***P=<0.0001; r=0.6507 (metaphase plate), r=0.6679 (cleavage furrow), r=-0.5292 (size ratio)). (**I**) Still images of NPCs seeded on box micropatterned fibronectin shapes undergoing mitosis in the absence or presence of ROCKi when the interphase nuclei is equatorially positioned prior to division (arrowheads, dashed arrows and dashed lines indicate cell front, retraction of cell front and cell-cell boundary, respectively). Schematics show aspect ratio (AR), front AR (fAR), no offset of the nuclei/metaphase plate/cleavage furrow, percentage retraction of the cell front and daughter size ratios. Plots show interphase and metaphase ARs for NPCs seeded on box fibronectin shapes in the presence or absence of ROCKi (n=18 control box cells, n=22 ROCKi box cells; two-tailed Mann-Whitney test, ***P=<0.0001). (**J**,**K**) Plots of either the offset of the metaphase plate and cleavage furrow from the midpoint of cell length in division (J) or of post-mitotic daughter size ratio (K) for NPCs seeded on micropatterned fibronectin box shapes in the presence or absence of ROCKi (sample sizes as in I; two-tailed Mann-Whitney test, ***P=0.0001). Data are mean ± s.d. (A,I,J and K). For I, scale bar, 10µm.

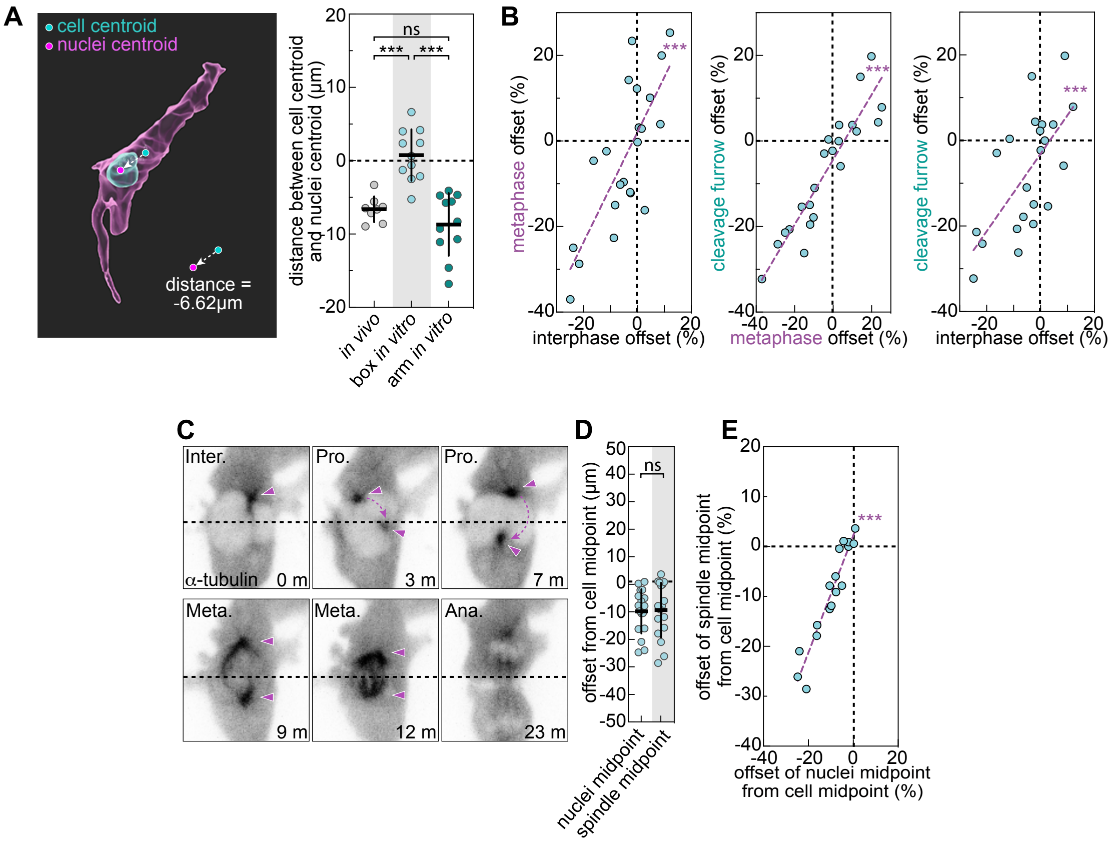

Fig. S7. Interphase nuclei positioning defines metaphase plate positioning in isomorphic division. (**A**) 3D volume rendering of a mosaic-labelled Cerulean-H2B- and lyn-mCherry-expressing zebrafish tEC in interphase with distance between the centroid of the cell volume and centroid of the nuclei volume indicated. Quantification of distance between the centroid of the cell volume and centroid of the nuclei volume for tECs in vivo, ECs in vitro seeded on seeded on micropatterned fibronectin box shapes, or ECs in vitro seeded on seeded on micropatterned fibronectin arm shapes (*n*=8 in vivo cells, *n*=11 in vitro box cells, *n*=11 in vitro arm cells; two-tailed unpaired t-test, ****P*=<0.0001, ns *P*=0.2169). (**B**) Plots comparing either offset of the nuclei in interphase from the tEC midpoint versus the offset of the metaphase plate (*r*=0.7698), offset of the metaphase plate from the tEC midpoint versus the offset of the cleavage furrow positioning (*r*=0.9229), or offset of the nuclei in interphase from the tEC midpoint versus offset of cleavage furrow positioning (*r*=0.6583 ;*n*=22 cells; two-tailed Pearson's correlation coefficient; ****P*=<0.0009). (**C**) Still images of a mosaic-labelled α-tubulin-GFP-expressing zebrafish tEC undergoing mitosis (Interphase (Inter.), Prophase (Pro.), Metaphase (Meta.), Anaphase (Ana.); magenta arrowheads, magenta dashed arrows and black dashed lines indicate centrosome/spindle pole positioning, centrosome movement and midpoint of the interphase nuclei, respectively). (**D**) Quantification of the offset of the midpoint of the interphase nuclei from the midpoint of the cell or the offset of the midpoint of the metaphase spindle from the midpoint of the cell in the same dividing tECs in vivo (*n*=17 cells; two-tailed unpaired t-test, ns *P*=0.8851). (**E**) Plot comparing the offset of the midpoint of the interphase nuclei from the midpoint of the cell versus the offset of the midpoint of the metaphase spindle from the midpoint of the cell in the same dividing tECs in vivo (*n*=17 cells; Pearson's correlation coefficient; ****P*=<0.0001; *r*=0.9520). Data are mean ± s.d. (A and D). For A and C, scale bars, 10µm.

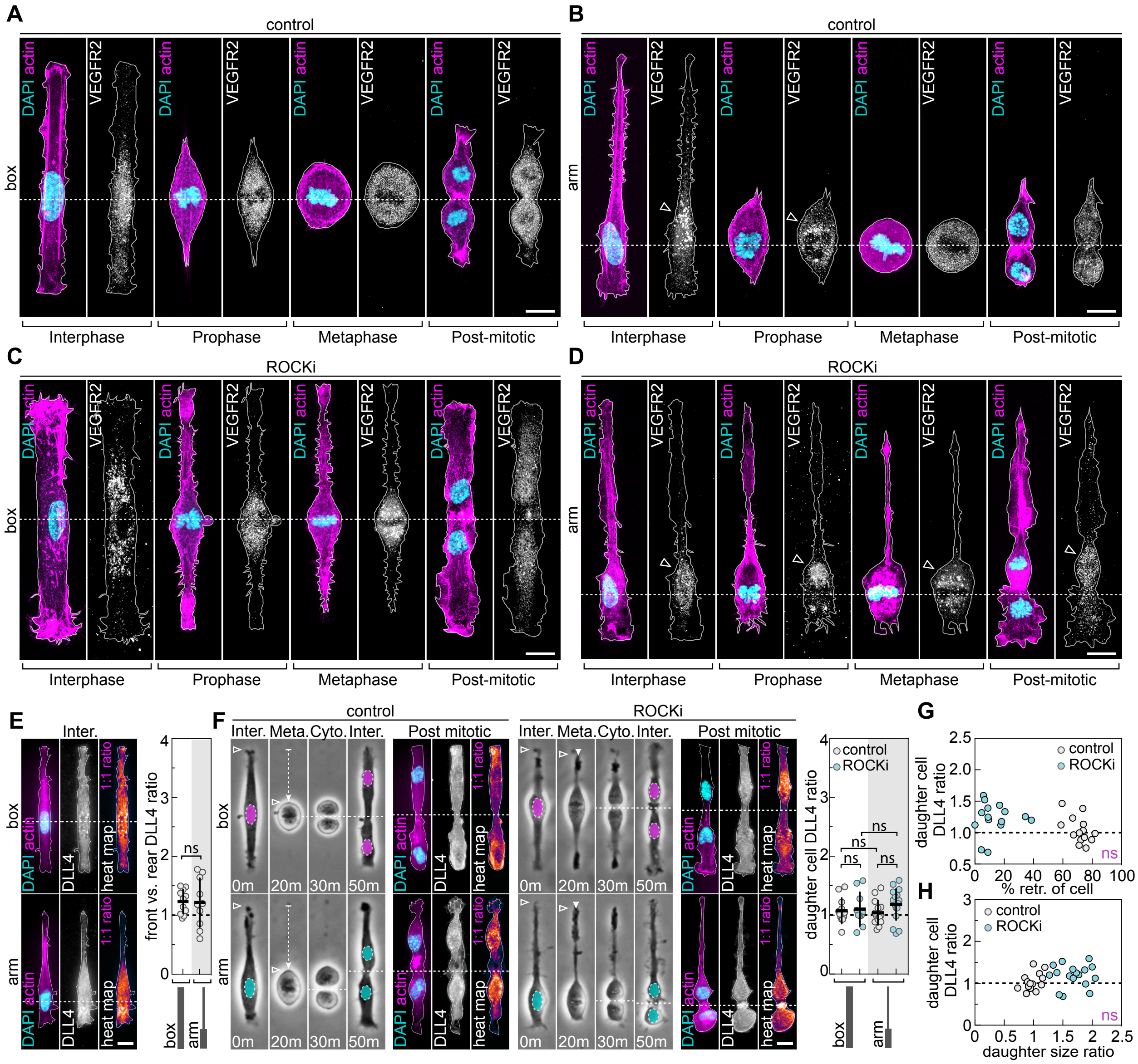

A
control
box
DAPI actin
VEGFR2
Interphase
Prophase
Metaphase
Post-mitotic
B
control
arm
C
ROCKi
D
ROCKi
E
Inter.
DLL4
heat map
1:1 ratio
front vs. rear DLL4 ratio
ns
F
control
ROCKi
Inter. Meta. Cyto. Inter.
Post mitotic
0m 20m 30m 50m
daughter cell DLL4 ratio
control
ROCKi
ns
G
daughter cell DLL4 ratio
% retr. of cell
H
daughter size ratio

Fig. S8. Interphase fate determinant polarity predicts mitotic partitioning in isomorphic division. (**A**-**D**) Images of interphase, prophase, metaphase and post-mitotic ECs seeded on either micropatterned fibronectin box shapes (A,C) or arm shapes (B,D) either in the absence (A,B) or presence (C,D) of ROCKi and immunostained for VEGFR2 (dashed line indicates either interphase nuclei position, prophase chromatin position, metaphase plate position or post-mitotic cell-cell boundary; arrowheads indicate asymmetric distribution of VEGFR2). (**F**) Images of interphase ECs seeded on micropatterned fibronectin shapes and immunostained for DLL4 (dashed line indicates nuclei position). Plot of the ratio of DLL4 fluorescence intensity forward versus rearward of EC nuclei (*n*=11 box cells, *n*=10 arm cells; two-tailed unpaired t-test, ns *P*=0.9035). (**F**) Representative still images of human ECs seeded on micropatterned fibronectin shapes undergoing mitosis in the presence or absence of ROCKi and immunostaining of these ECs for DLL4 immediately after division (arrrowheads, dashed arrows and dashed lines indicate cell front, retraction of cell front and cell-cell boundary, respectively; nuclei are pseudocolored). Plot of the ratio of DLL4 fluorescence intensity between daughter ECs (*n*=12 control box cells, *n*=10 ROCKi box cells, *n*=14 control arm cells, *n*=16 ROCKi arm cells; Unpaired ANOVA and Tukey's multiple comparison test; ns *P*=>0.3964). (**G**,**H**) Plots comparing either the percentage retraction of the cell front (G) or daughter EC size ratio (H) versus the ratio of DLL4 fluorescence intensity between daughters for control and ROCKi-treated ECs seeded on micropatterned fibronectin arm-shapes (black lines indicate DLL4 symmetry; sample sizes as in F; two-tailed Pearson's correlation coefficient; ns *P*=>0.0663). Data are mean ± s.d. (E and F). For A,B,C,D,E and F, scale bars, 10μm.

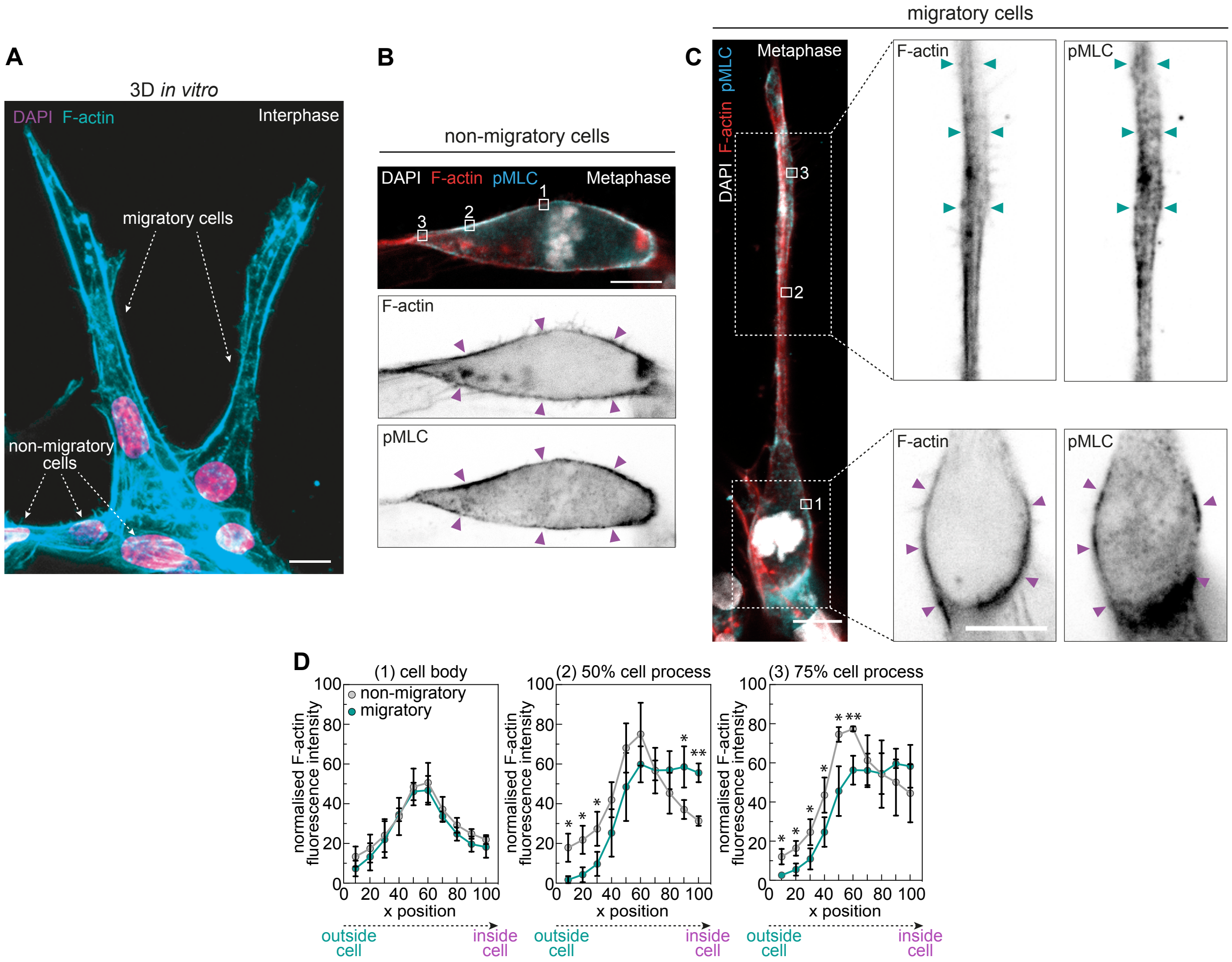

Fig. S9. Isomorphic divisions in 3D culture exhibit spatial differences in actomyosin remodelling. (**A-C**) Representative images of interphase (A) or metaphase (B,C) human ECs in 3D fibrin gel bead assays and treated with rhodamine-phalloidin and immunostained for phospho-Myosin Light Chain (pMLC; B,C). ECs are categorized as either migratory ECs that lead new branches and are highly protrusive in interphase (A,C) or non-migratory ECs that remain attached to the bead and are less elongated in interphase (A,B). Mitotic non-migratory ECs robustly recruit cortical F-actin and pMLC (B; magenta arrowheads). Likewise, mitotic protrusive migratory ECs robustly recruit cortical F-actin and pMLC to the cell body (C; magenta arrowheads), but exhibit reduced recruitment of cortical F-actin and pMLC to elongated motile cell processes (C; cyan arrowheads). (**D**) Plots of the cortical intensity of either F-actin at either the cell body, 50% along the cell process, or 75% along the cell process (positions 1, 2 and 3, respectively, in panels B and C) for non-migratory cell (grey line) and migratory cells (cyan line) in mitosis (n=3 cells; two-tailed unpaired t-test, **P=<0.0082, *P=0.0497). Data are mean ± s.d. (D). For A, B and C scale bars, 10μm.

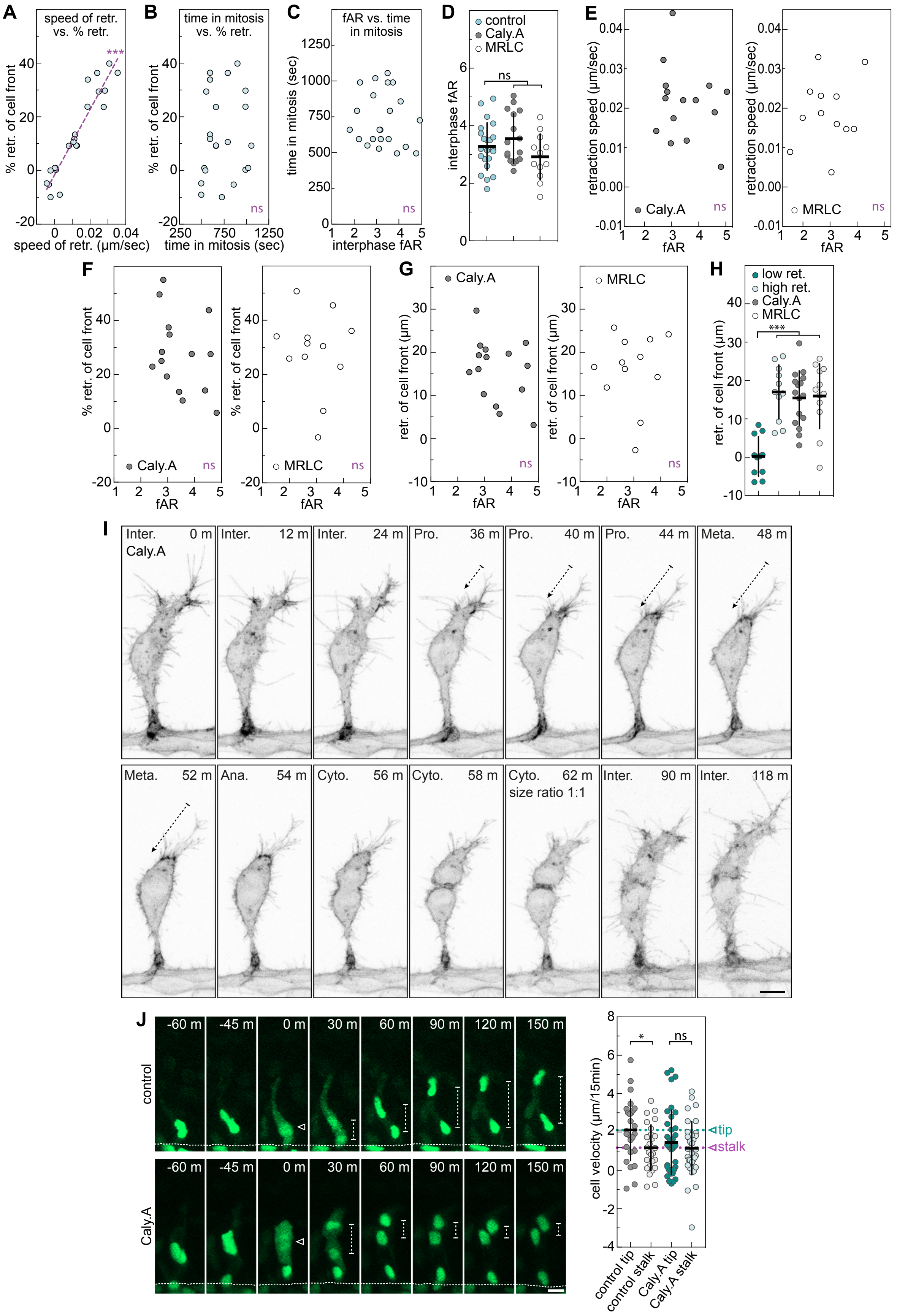
A
speed of retr. vs. % retr.
% retr. of cell front
speed of retr. (µm/sec)
***
B
time in mitosis vs. % retr.
% retr. of cell front
time in mitosis (sec)
ns
C
fAR vs. time in mitosis
time in mitosis (sec)
interphase fAR
ns
D
control
Caly.A
MRLC
interphase fAR
ns
E
retraction speed (µm/sec)
fAR
Caly.A
MRLC
ns
F
% retr. of cell front
fAR
Caly.A
MRLC
ns
G
retr. of cell front (µm)
fAR
Caly.A
MRLC
ns
H
low ret.
high ret.
Caly.A
MRLC
retr. of cell front (µm)
***
I
Inter. Caly.A 0 m
Inter. 12 m
Inter. 24 m
Pro. 36 m
Pro. 40 m
Pro. 44 m
Meta. 48 m
Meta. 52 m
Ana. 54 m
Cyto. 56 m
Cyto. 58 m
Cyto. 62 m size ratio 1:1
Inter. 90 m
Inter. 118 m
J
control
Caly.A
-60 m
-45 m
0 m
30 m
60 m
90 m
120 m
150 m
cell velocity (µm/15min)
*
ns
tip
stalk
control tip
control stalk
Caly.A tip
Caly.A stalk

Fig. S10. Modulation of mitotic actomyosin perturbs isomorphic division in vivo. (**A**) Plot comparing speed of membrane process retraction (retr.) versus the percentage retraction of the cell front during tEC mitosis in vivo (*n*=22 cells; two-tailed Pearson's correlation coefficient; ****P*=<0.0001; *r*=0.9430). (**B**) Plot comparing the time spent in mitosis versus the percentage retraction of the cell front during tEC division (*n*=22 cells; two-tailed Pearson's correlation coefficient; ns *P*=0.5812). (**C**) Plot comparing the front aspect ratio (fAR) of tECs in interphase versus the time spent in mitosis (*n*=22 cells; two-tailed Pearson's correlation coefficient; ns *P*=0.6061). (**D**) Quantification of the fAR of interphase tECs either in control conditions, upon treatment with calyculin A (Caly.A) or upon expression of MRLC-GFP (MRLC; *n*=22 control cells, *n*=16 Caly.A cells, *n*=12 MRLC cells; Unpaired ANOVA and Tukey's multiple comparison test; ns *P*=>0.1237). (**E**,**F**,**G**) Plots comparing the fAR of interphase tECs versus either the speed of membrane process retraction (E), the percentage retraction of the cell front (F), or the amount of retraction of the cell front in µm (G) upon treatment with Caly.A or upon expression of MRLC-GFP. Plots reveal a loss of correlation (*n*=16 Caly.A cells, *n*=12 MRLC cells; two-tailed Pearson's correlation coefficient; ns *P*=>0.0975). (**H**) Quantification of the amount of retraction of the cell front in µm in either Wt tECs (split into the 50% highest retracting or 50% lowest retracting cells), Caly.A-treated tECs or MRLC-GFP-expressing tECs (*n*=22 Wt cells, *n*=16 Caly.A cells, *n*=12 MRLC cells; Unpaired ANOVA and Tukey's multiple comparison test; ****P*=<0.0001, all statistical comparisons are indicated by brackets). (**I**) Still images of a lyn-mCherry-expressing zebrafish tEC undergoing mitosis in the presence of Caly.A (Interphase (Inter.), Prophase (Pro.), Metaphase (Meta.), Anaphase (Ana.), Telophase (Telo.), Cytokinesis (Cyto.); dashed arrows indicate retraction of cell front). (**J**) Time-lapse images of nlsEGFP-expressing EC nuclei in sprouting vessels prior to, during and after tip cell division in control or Caly.A-treated embryos (arrowheads indicate mitosis; brackets indicate distance between both daughter cells; dotted line indicates position of parental vessel). Plot shows quantification of the velocity of tip or stalk cell daughters in control or Caly.A-treated embryos (cyan and magenta dotted lines indicate the motility of normal tip and stalk cells, respectively; *n*=26 Wt cells, *n*=36 Caly.A cells; two-tailed unpaired t-test; **P*=0.0213, ns *P*=0.4157). Data are mean ± s.d. (D,H and J). For I and J, scale bars, 10µm.

Movie S1. Time lapse live imaging of a zebrafish tEC expressing Cerulean-H2B (magenta) and lyn-mCherry (cyan) undergoing mitosis, shown in Fig. 1A. Images were acquired every 2 minutes and max-intensity projected images converted into a 5 fps movie. Quantification shown in Fig. 1D. Scale bar 10μm.

Movie S2. Time lapse live imaging of a human EC in 2D culture undergoing mitosis, shown in Fig. 1C. Widefield images acquired every 5 minutes and converted to a 5 frames per second (fps) movie. Quantification shown in Fig. 1D. Scale bar 20μm.

Movie S3. Time lapse live imaging of a zebrafish tEC expressing lyn-mCherry (cyan) with high fAR undergoing mitosis, shown in Fig.1E (top). Images were acquired every 66 seconds and max-intensity projected images converted into a 5 fps movie. Arrowheads indicate cell front. Quantification shown in Fig. 1E (right) and 1G. Scale bar 10μm.

Movie S4. Time lapse live imaging of a zebrafish tEC expressing lyn-mCherry (cyan) with low fAR undergoing mitosis, shown in Fig.1E (bottom). Images were acquired every minute and max-intensity projected images converted into a 5 fps movie. Arrowheads indicate cell front; arrow indicates retraction of cell front. Quantification shown in Fig. 1E (right) and 1G. Scale bar 10μm.

Movie S5. Time lapse live imaging of a human EC in 2D culture undergoing mitosis on micropatterned line of fibronectin, shown in Fig. 1F (top). Widefield images acquired every 5 minutes and converted to a 5 frames per second (fps) movie. Arrowheads indicate cell front; arrow indicates retraction of cell front. Quantification shown in Fig. 1F (right) and1G. Scale bar 10μm.

Movie S6. Time lapse live imaging of a human EC in 2D culture undergoing mitosis on micropatterned line of fibronectin, shown in Fig. 1F (bottom). Widefield images acquired every 5 minutes and converted to a 5 frames per second (fps) movie. Arrowheads indicate cell front; arrow indicates retraction of cell front. Quantification shown in Fig. 1F (right) and 1G. Scale bar 10μm.

Movie S7. Time lapse live imaging of a zebrafish trunk neural crest cell expressing H2B-mCherry (magenta) and GLYPI-EGFP (cyan) undergoing mitosis, shown in Fig. 1I (top). Images were acquired every 5 minutes and max-intensity projected images converted into a 5 fps movie. Arrowheads indicate cell front. Quantification shown in Fig. 1H and 1J. Scale bar 10μm.

Movie S8. Time lapse live imaging of a zebrafish PLLp cell expressing lyn-EGFP (white) undergoing mitosis (pseudo-coloured cyan and daughters in pink and yellow), shown in Fig. 1I (bottom). Images were acquired every 2 minutes and max-intensity projected images converted into a 5 fps movie. Quantification shown in Fig. 1H and 1J. Scale bar 10μm.

Movie S9. Time lapse live imaging of human ECs in 2D culture undergoing mitosis on micropatterned box (left) and arm (right) patterns of fibronectin, shown in Fig. 2D (left). Widefield images acquired every 5 minutes and converted to a 5 frames per second (fps) movie.

Arrowheads indicate cell front; arrows indicate retraction of cell front. Quantification shown in Fig. 2E,F,G and H. Scale bar 10μm.

Movie S10. Time lapse live imaging of human ECs in 2D culture undergoing mitosis on micropatterned box (left) and arm (right) patterns of fibronectin and treated with ROCKi, shown in Fig. 2D (right). Widefield images acquired every 5 minutes and converted to a 5 frames per second (fps) movie. Arrowheads indicate cell front; arrows indicate retraction of cell front. Quantification shown in Fig. 2E,F,G and H. Scale bar 10μm.

Movie S11. Time lapse live imaging of a zebrafish tEC expressing Cerulean-H2B (magenta) and lyn-mCherry (cyan) undergoing mitosis, shown in Fig. 4E. Images were acquired every minute and max-intensity projected images converted into a 5 fps movie. Solid arrow heads mark the mid-point and open arrow heads the widest point of the cell. Quantification shown in Fig. 4F. Scale bar 10μm.

Movie S12. Time lapse live imaging of a zebrafish tEC mosaically expressing Lifeact-GFP (cyan) with high fAR undergoing mitosis, shown in Fig. 6A. Images were acquired every 66 seconds and max-intensity projected images converted into a 5 fps movie. White arrowheads indicate cell front; red arrow heads indicate growing filopodia. Quantification shown in Fig. 6B-D. Scale bar 10μm.

Movie S13. Time lapse live imaging of a zebrafish tEC mosaically expressing Lifeact-GFP (cyan) with low fAR undergoing mitosis, shown in Fig. 6F. Images were acquired every 66 seconds and max-intensity projected images converted into a 5 fps movie. Arrowheads indicate cell front; arrows indicate retraction of cell front. Quantification shown in Fig. 6G-I. Scale bar 10μm.

Movie S14. Time lapse live imaging of a zebrafish tEC mosaically expressing MRLC-GFP (cyan) undergoing mitosis, shown in Fig. 6L (top). Images were acquired every 66 seconds and max-intensity projected images converted into a 5 fps movie. Arrowheads indicate cell front; arrow indicates retraction of cell front. Quantification shown in Fig. 6M,N and O. Scale bar 10μm.

Movie S15. Time lapse live imaging of a zebrafish tEC expressing lyn-mCherry (cyan) treated with calyculin A undergoing mitosis, shown in Fig. 6L (bottom). Images were acquired every 66 seconds and max-intensity projected images converted into a 5 fps movie. Arrowheads indicate cell front; arrow indicates retraction of cell front. Quantification shown in Fig. 6M,N and O. Scale bar 10μm.
